# Dyke apertures record stress accumulation during sustained volcanism

**DOI:** 10.1038/s41598-020-74361-w

**Published:** 2020-10-15

**Authors:** Samuel T. Thiele, Alexander R. Cruden, Steven Micklethwaite, Andrew P. Bunger, Jonas Köpping

**Affiliations:** 1 School of Earth, Atmosphere and Environment, Monash University, Melbourne, 3800 Australia; 2 Helmholtz Institute Freiberg for Resource Technology, Helmholtz-Zentrum Dresden-Rossendorf, 09599 Freiberg, Germany; 3 Department of Civil and Environmental Engineering, University of Pittsburgh, Pittsburgh, PA 15269 USA; 4 Department of Chemical and Petroleum Engineering, University of Pittsburgh, Pittsburgh, PA 15269 USA

**Keywords:** Natural hazards, Solid Earth sciences

## Abstract

The feedback between dyke and sill intrusions and the evolution of stresses within volcanic systems is poorly understood, despite its importance for magma transport and volcano instability. Long-lived ocean island volcanoes are crosscut by thousands of dykes, which must be accommodated through a combination of flank slip and visco-elastic deformation. Flank slip is dominant in some volcanoes (e.g., Kilauea), but how intrusions are accommodated in other volcanic systems remains unknown. Here we apply digital mapping techniques to collect > 400,000 orientation and aperture measurements from 519 sheet intrusions within Volcán Taburiente (La Palma, Canary Islands, Spain) and investigate their emplacement and accommodation. We show that vertically ascending dykes were deflected to propagate laterally as they approached the surface of the volcano, forming a radial dyke swarm, and propose a visco-elastic model for their accommodation. Our model reproduces the measured dyke-aperture distribution and predicts that stress accumulates within densely intruded regions of the volcano, blocking subsequent dykes and causing eruptive activity to migrate. These results have significant implications for the organisation of magma transport within volcanic edifices, and the evolution and stability of long-lived volcanic systems.

## Introduction

Magma plumbing systems comprise temporally and spatially interconnected dykes, sills and magma chambers that exert fundamental controls on volcanic behaviour^1^. They influence and reflect edifice processes, and so provide a record of volcano dynamics and long-term evolution that is essential for the development of predictive models. Different volcanoes exhibit a range of magma plumbing styles due to variations in geological setting. For example, the mechanical properties of the volcanic basement, and flexure of the underlying lithosphere, can exert a first-order control on stresses within and beneath volcanic edifices, and hence the organisation of their magma plumbing systems^2,3^. Similarly, magma plumbing systems have been suggested to be influenced by topographic stresses^4–6^, uplift due to the emplacement of magma at depth^7,8^, edifice instability^3,9,10^, remote tectonic stresses^11,12^ and basement structures^13^.

**Figure 1:**
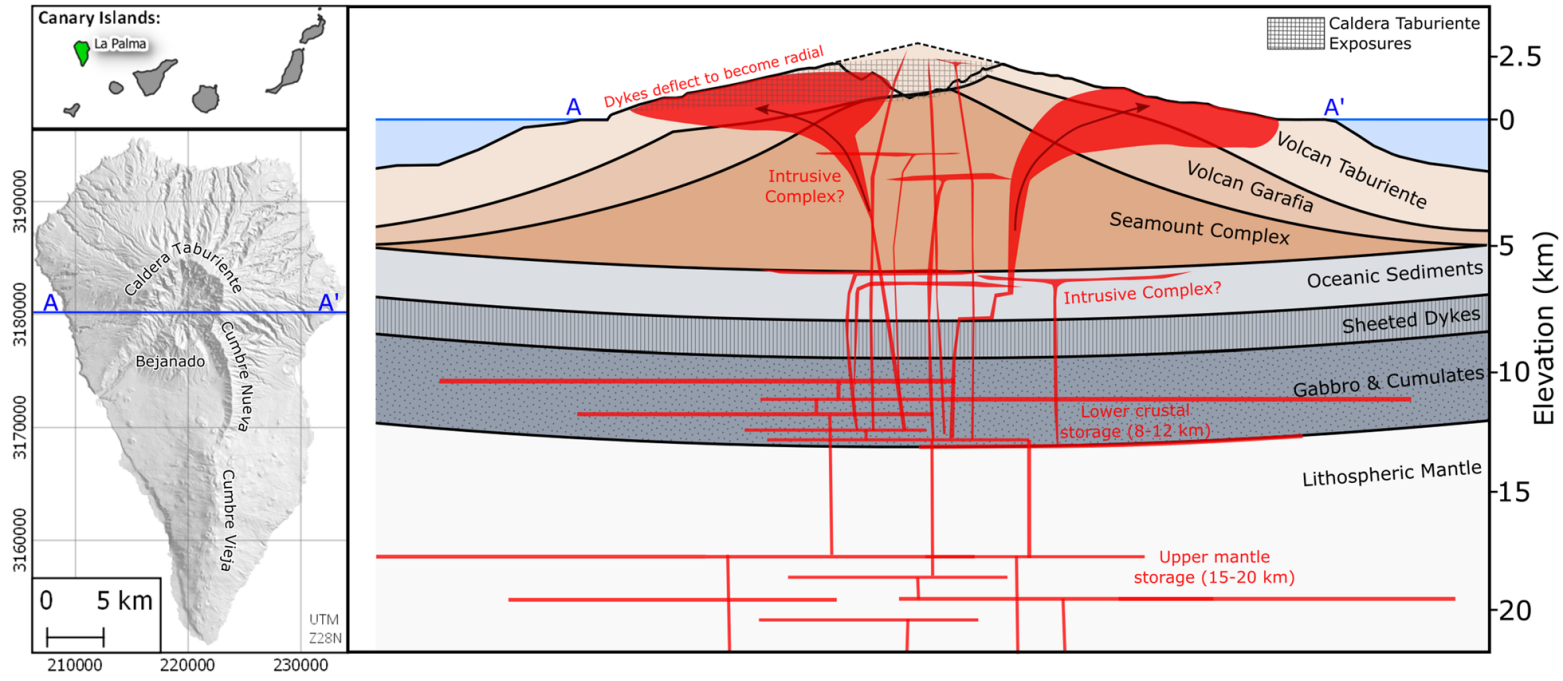
Location and prominent topographic features of La Palma, Canary Islands. Schematic cross section A–A′ through Volcán Taburiente is based on various geobarometry studies^20,21^. Deflection of dykes in the upper parts of the edifice to propagate laterally is inferred from results of this study. Map created using QGIS 2.18 (https://www.qgis.org).

In this contribution we investigate the mechanisms that control and accommodate dyke injections in the shallow plumbing system of Volcán Taburiente, which forms the northern part of La Palma (Canary Islands; Fig. 1). This area is of interest as many thousands of intrusions^1,11,14^ are exceptionally well exposed along spectacular cliff sections, and because these intrusions may record the self-organisation of a radial magma plumbing system into a discrete rift zone^15^. The eventual collapse of the edifice after ca. 400 kyr of activity^16^ could also be linked to this plumbing system reorganisation.

### Geological setting

The Canary Islands are constructed on slow moving (< 2 cm/year) Jurassic oceanic crust west of the coast of Morocco. As a result, volcanic islands in this region tend to be long-lived compared to e.g., Hawaiian volcanoes, due to lower plate-velocities, and they undergo limited subsidence because of the stiffness of the underlying oceanic crust^17^. The intraplate setting of the Canary Islands and their distance from Atlantic mid-ocean ridge spreading centres also results in low horizontal differential stresses compared to other tectonic settings^11,18^.

It is broadly accepted that magmatism at La Palma (and the other Canary Islands) results from an upwelling mantle plume that triggers mantle melting through decompression and the addition of heat^17^. However, magma production rates are much lower than at other hotspots^17,19^, and as a result many of the islands (including La Palma) do not appear to host long-lived shallow magma reservoirs. This hypothesis is supported by pyroxene phenocryst and melt inclusion geobarometry^20,21^ and geophysical observations from eruptions at the neighbouring El Hierro in 2011^22^.

Instead, eruptions on La Palma appear to be fed by magma that accumulated and crystallised pyroxene and olivine within deep, long-lived reservoirs in the upper mantle (~ 20–30 + km depth) before being emplaced for a much shorter period of time (weeks to months) in the uppermost mantle or lower crust (10–15 km depth), and finally migrating upwards along rapidly propagating dykes to erupt (Fig. 1).

As is typical of ocean island volcanoes, each of the Canary Islands is constructed from several distinct volcanic edifices. La Palma consists of an uplifted Pliocene seamount (~ 4–2 Ma) overlain by a succession of sub-aerial edifices: Garafia (~ 2–1.2 Ma), Taburiente (1.2–0.56 Ma), Bejenado (0.56–0.49 Ma), and Cumbre Vieja (0.56 Ma to present)^16^. Lavas and pyroclastic products from Volcán Taburiente cover most of the north of the island, with Garafia and seamount complex rocks exposed only along the bottom of deeply incised ravines. The southern part of the island is formed by the currently active Cumbre Vieja volcano, a N–S oriented ridge lined with Holocene scoria cones and lava flows^16^.

Each of these volcanic edifices are separated by significant erosional unconformities, generally related to large edifice collapse events^15^. The Garafia edifice is thought to have collapsed to the south-west at ~ 1.2 Ma, forming a large depression that was rapidly filled by Volcán Taburiente^11,17^. From ~ 0.8 Ma Taburiente volcanism appears to have migrated southwards, extending the edifice’s southern flank and forming an elongate, N-S oriented ridge. This ridge collapsed towards the west at ~ 0.56 Ma, after which post-collapse volcanism rapidly formed the Bejenado edifice^16^. However volcanism continued to migrate southwards, leaving the collapse escarpment (now defined by the Cumbre Nueva ridge^15^) unfilled and forming the currently active Cumbre Vieja edifice in the south of the island.

Dykes in the shallow parts of the Taburiente edifice have radial orientations^1,11,14^, indicating that the least compressive stress σ_3_ is oriented circumferentially around a central point. Circumferential orientations of σ_3_ can be caused by a source of pressure at the swarm centre (e.g., a magma chamber) or by a radially decreasing topographic load (as is typically imposed by a volcanic edifice), although topographic stresses tend to dominate at shallow depths^23^. Similar radial dyke swarms have been reported from Mt Somma/Vesuvio (Italy)^24^, Summer Coon (USA)^25^, Oki-Dozen (Japan)^26^ and Lyttleton (New Zealand)^27^.

While dykes that are injected into elongate rift zones such as those in Hawaii can be accommodated by lateral flank displacement^28^, the accommodation mechanism for radial swarms is less clear. With this in mind, we present data on the orientation and thickness of dykes within the Volcán Taburiente, quantify the bulk-strain they induced, and consider their influence on intra-edifice stresses and the evolution of the magma plumbing system.

## Results

### Orientation and thickness

Erosion of the Cumbre Nueva collapse scarp has incised deep into Volcán Taburiente to form an arcuate series of ~ 1 km high cliffs known as Caldera Taburiente^15,29^ (Fig. 1). We have taken advantage of this landscape to map the spectacularly exposed shallow magma plumbing system in unprecedented detail using 14 unmanned aerial vehicle (UAV) surveys conducted over ~ 2–50 hectare areas. Emerging three-dimensional (3-D) digital mapping techniques^30,31^ were then applied to extract > 400,000 orientation and thickness measurements from 519 sheet intrusions.

As expected, these measurements highlight the generally radial orientations of the intrusions. We constrain the focal point of the radial dyke swarm using strike measurements and a maximum likelihood estimator (see Supplementary Method), which delineates an area in the southern part of Caldera Taburiente ~ 1.5 km north of Bejenado (see Supplementary Fig. S1). Surveys from the northern side of the Caldera (Las Pareditas, Risco Liso, Hoyo Verde and Los Cantos) also suggest a population of thicker and somewhat shallower dipping intrusions striking NW and crosscut by the radial dykes (see Supplementary Figs. S1, S2).

Many sheet intrusions in Caldera Taburiente have geometries that suggest they propagated laterally. These include basal terminations, step-overs and broken bridge structures with shallow dipping axes (see Supplementary Fig. S2). Field observations of stretched vesicles and striated chilled margins associated with strongly aligned plagioclase flow fabrics^32^ indicate flow lineations that plunge gently (0°–40°) both towards and away from the dyke swarm focal point (see Supplementary Fig. S1). As such, we interpret that dykes ascending vertically from below Volcán Taburiente were deflected to propagate laterally and radially as they approached the surface (Fig. 1).

**Figure 2:**
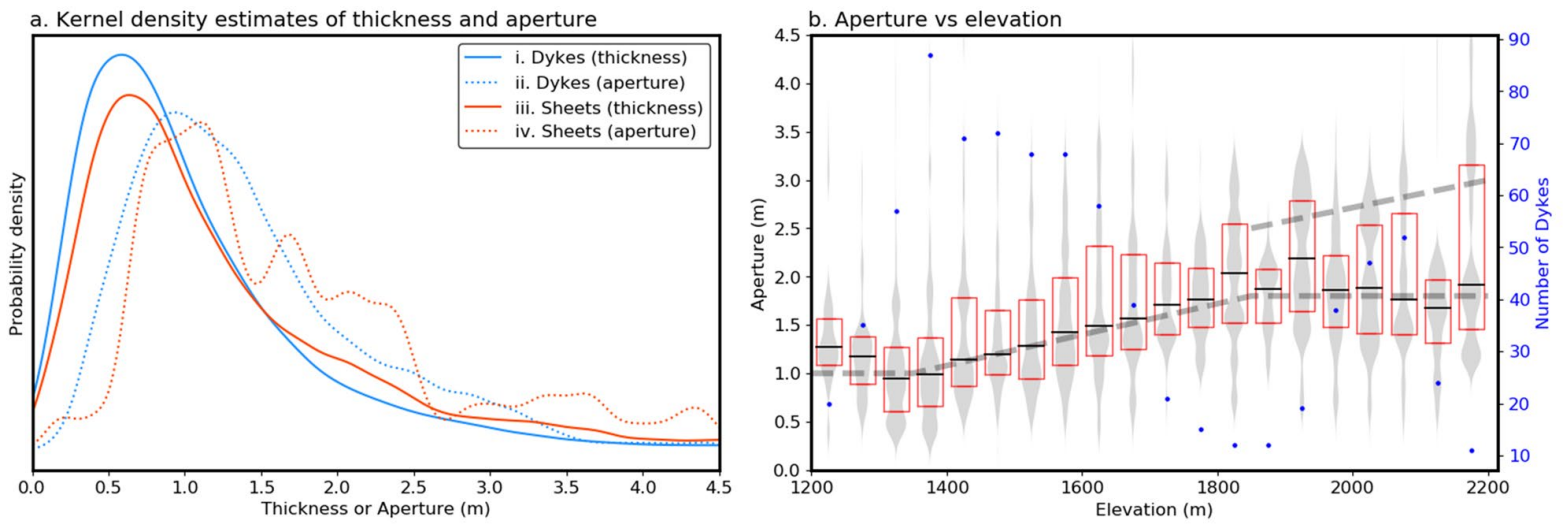
Kernel density estimates (**a**) describing the thickness and aperture (defined as the 75th to 90th percentiles of thickness measurements for each dyke) of all dykes (i, ii) and inclined sheets (iii, iv). Thickness measurements were also divided into 50 m high bins (**b**) based on their elevation and the interquartile range (red lines), median (black line) and a kernel density estimate of the underlying distribution (grey) plotted. This shows a clear increase in thickness between ~ 1400 and 1800 m above sea level. No clear trend in thickness can be seen above this altitude, although unusually thick dykes appear to become more common.

A large range of thicknesses were measured for both dykes (dip > 45°) and inclined sheets (dips < 45°, including true sills). Measurements from dykes have a mode thickness of ~ 0.6 m and a long-tailed distribution, extending to ~ 5 m (Fig. 2a). To avoid biases due to measurements near dyke tips, a ‘maximum aperture’ dataset was created by removing measurements below the 75th percentile and those above the 90th percentile (assumed to reflect erroneous measurements or abnormally thick dyke sections). For clarity, we use the term ‘thickness’ to describe the collection of all measurements and ‘aperture’ to refer to the estimates of each intrusion’s maximum thickness. The aperture measurements have a mode of ~ 1 m and a long-tailed distribution similar to the thickness data (Fig. 2a).

The thickness and aperture of inclined sheets are similar to the dykes, although they tend towards higher values (Fig. 2a) with modes of ~ 0.6 and 1.25 m respectively. Unlike the dykes, however, the aperture distribution of inclined sheets contains anomalously thick subpopulations (> 30 m thick, e.g. Supplementary Fig. S2). This irregularity could be because fewer inclined sheets were sampled, but might also suggest that the apertures of sills and other shallow-dipping intrusions are limited by different factors to dykes.

A striking relationship can be observed between intrusion altitude, a rough proxy for its depth of formation, and thickness. Below ~ 1400 m above sea level, the intrusions have a median thickness of ~ 1 m, but by ~ 1800 m this has increased to nearly 2 m, where it appears to plateau (Fig. 2b). Based on a paleo-altitude estimate of ~ 2500 m around the rim of present-day Caldera Taburiente (the current elevation of its highest point), and assuming that most of the dykes formed when the edifice was similar to its current size, this region of increasing dyke thickness corresponds to below surface paleo-depths of ~ 1100 to 700 m. Above ~ 1800 m altitude (< 700 m paleo-depth) the frequency of > 2.5 m thick dykes also appears to increase (Fig. 2b).

### Excess magma pressure

The ratio between dyke aperture and length has been used by several authors to estimate excess pressure driving dyke emplacement^26,33–35^, where excess pressure is the difference between total magma pressure and the normal stress resisting initial dyke opening. We have applied this method to our dataset by selecting 52 dykes that are exposed in their entirety in the UAV surveys, such that their tip-to-tip ‘span’ could be measured. Assuming the dykes propagated sub-horizontally, as suggested previously, these span measurements were projected onto a vertical plane to give measurements of the dyke height (*h*). Excess pressure (*P*_*excess*_) was then estimated using the aperture (*a*) measurements; assuming that the dykes are much longer than they are high and vertical pressure variations are negligible, *P*_*excess*_ can be related to *a* and *h* using the plane-strain solution to a pressurised elliptical crack^33,35^:

1 $${P}_{excess}= \frac{aE}{2h(1-{v}^{2})}$$

This excess pressure estimate will vary proportionally to the elastic properties of the volcanic edifice. The bulk Young’s modulus (*E*) is rather difficult to ascertain, and in this context presumably depends on the ratio of compliant pyroclastic material to stiff basalt flows. Values of 1 to 5 GPa have been used by previous authors for studies on basaltic volcanoes^25,33,34^, and hence we evaluate excess pressure over this range. Poisson’s ratio (*v*) was kept fixed at 0.25.

**Figure 3:**
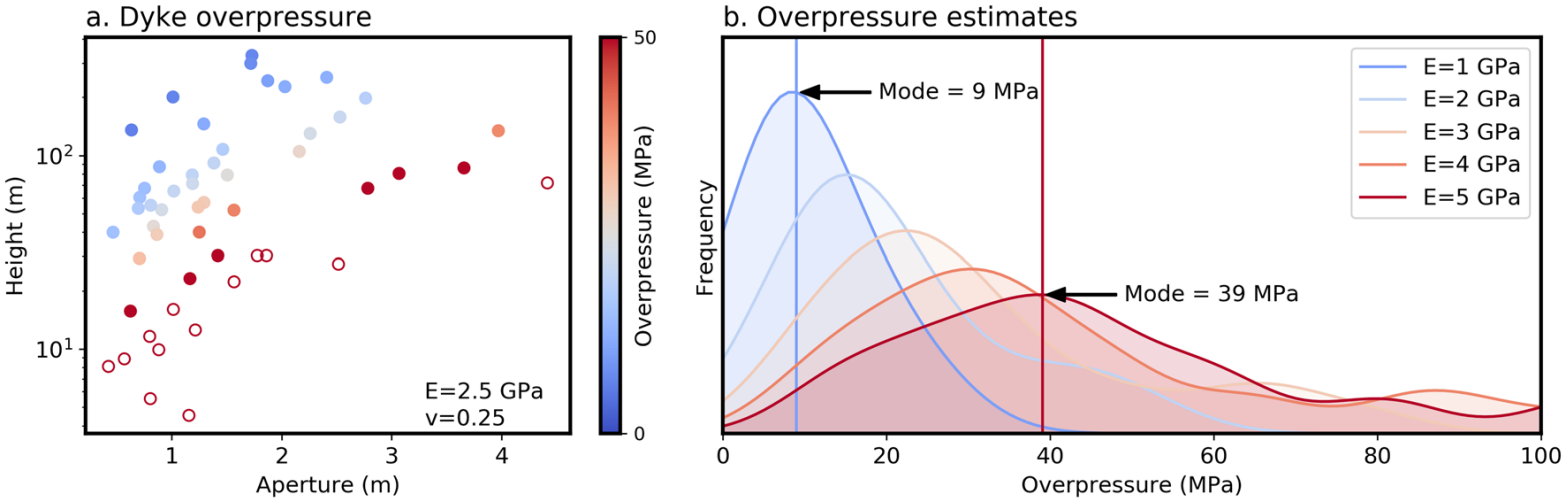
Height and aperture of the 52 dykes for which measurements could be obtained (**a**), coloured using overpressure estimates calculated using Eq. (1) and a Young’s modulus (E) and Poisson’s ratio (v) of 2.5 GPa and 0.25 respectively. The top 25% of estimates were considered to be outliers (unfilled circles) as they result from dykes with unusually small length-aperture ratios. Kernel density estimates of the calculated excess pressure using different Young’s moduli highlight significant uncertainty and/or variation (**b**), although they suggest that typical values between 10 and 60 MPa seems plausible. Note that outliers (top 25% of calculated excess pressure) were excluded during the kernel density estimation.

The results (Fig. 3) show substantial variation in estimated excess pressure, probably because (1) propagation directions may not have been strictly horizontal, meaning the dyke ‘height’ will be under- or over-estimated, (2) solidified apertures may not represent the fracture aperture during dyke propagation, and (3) a range of actual magma pressure is likely. Regardless of these limitations, the results indicate excess pressures of ~ 10 to 60 MPa, similar to estimates from other studies^25,33,34^.

### Induced strain

**Figure 4:**
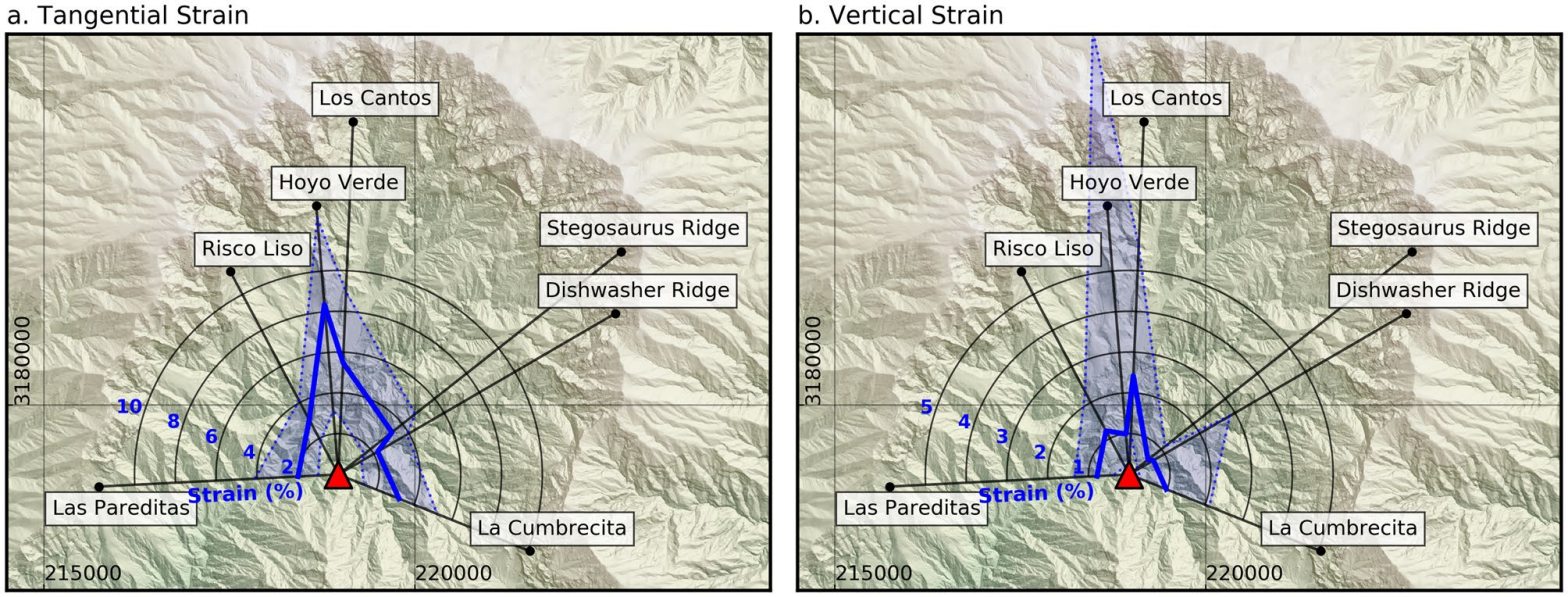
Map showing the median (solid line), 10th and 90th percentiles (dotted lines) of tangential (**a**) and vertical (**b**) strain estimated using 1 m spaced scan lines extracted from each digital outcrop model. These show a general tangential strain of ~ 2–4% through much of the caldera and substantially higher values (6–10%) in the north. A similar pattern is observed for vertical strain, although the net vertical extension is much less (~ 1–2%). Data from Los Andenes has been omitted from this analysis due to the limited number of intrusions at that site. Note the difference in the strain plot scale between (**a**,**b**).

The continuity of exposure in Caldera Taburiente allowed us to estimate the vertical and tangential strain induced by the intrusions in Volcán Taburiente, using a cylindrical coordinate system with its origin at the previously described maximum likelihood radial centre. Tangential strain estimates range between 1 and 10%, with maximum values observed in the north of the caldera (Fig. 4a). An increased number of intrusions in this area is noticeable both in the field and in aerial imagery, suggesting that these results are reasonable. Vertical strain (Fig. 4b) follows a similar but weaker trend, suggesting relatively modest endogenous growth from shallow intrusions (1–2%). The large spike in the 90th percentile of estimated vertical strain at the Hoyo Verde site results from a single very thick (> 30 m) inclined sheet (Supplementary Fig. S2), and is probably only a local effect.

### Strain accommodation mechanisms

This strain must have been accommodated by a combination of (1) accumulated elastic stresses in the edifice, (2) ductile and plastic deformation such as compaction and faulting, and/or (3) flank movement along a basal detachment or ductile layer. Mechanisms (1) and (2) can be combined in a Maxwell visco-elastic model that estimates the edifice stresses that result from intrusions in the absence of a basal detachment and associated flank slip.

First, we define a polar coordinate system with *r* and *θ* corresponding to the radial distance and rotation (strike direction) relative to the focal point of the dykes, respectively (Supplementary Fig. S3). Assuming that radial and vertical stresses are transmitted to the volcano’s free surface (σ_r_ = σ_z_ = 0), stress caused by the injection of the radial dykes (σ_θ_) will be uniaxial in the circumferential direction. To avoid unreasonably large strains near the centre of the dyke swarm, we also assume that ascending dykes are deflected towards areas of lowest stress and so, as an ensemble, induce approximately constant circumferential stress. Thus, the rate of dyke-injections (*f*_*d*_) varies in proportion with 2π*r* such that the rate of circumferential strain (ε̇_θ_) is spatially uniform (Supplementary Fig. S3). This is convenient as it makes our choice of *r* arbitrary (we use 5 km). Similar accommodation-stress induced migration of volcanism has been demonstrated by Derrien and Taisne^36^, who simulated repeated dyke injection into an analogue volcano.

Based on these assumptions, we can relate ε̇_θ_ with σ_θ_ and the edifice’s bulk properties (shear modulus *G* and viscosity µ) using the Maxwell visco-elastic constitutive equation (see Supplementary Method for derivation):

2 $$\dot{{\varepsilon }_{\theta }}= \frac{1}{2G}\dot{{\sigma }_{\theta }}+ \frac{1}{\mu }{\sigma }_{\theta }$$

Strain rate ε̇_θ_ can be related to the frequency *f*_*d*_ at which dykes intrude across a given circumference (defined by radius *r*) and their aperture *a*, rewriting Eq. (2) as:

3 $${f}_{d}\frac{a}{2\pi r}= \frac{1}{2G}\dot{{\sigma }_{\theta }}+ \frac{1}{\mu }{\sigma }_{\theta }$$

Using Eq. (1), we can also relate dyke apertures to the excess magma pressure, which we here define as some initial excess pressure *P*_0_ minus the accumulated accommodation stresses σ_θ_. Substituting into Eq. (3) and re-arranging into a conventional form for a first-order, linear ordinary differential equation we get:

4 $$\dot{{\sigma }_{\theta }}+2G\left(\frac{1}{\mu }+k\right){\sigma }_{\theta } = 2Gk{P}_{0},$$

noting that:

5 $$k= {f}_{d}\frac{2h(1-{v}^{2})}{2\pi rE}.$$

This differential equation (Eq. (6)) can be solved analytically to give the accommodation stress as a function of time *t* (assuming that the initial σ_θ_ = 0), namely:

6 $${\sigma }_{\theta } = k \left(\frac{{P}_{0}}{\frac{1}{\mu }+k}-\frac{{P}_{0}}{\frac{1}{\mu }+k}{e}^{-2G\left(\frac{1}{\mu }+k\right)t}\right).$$

Values for *G* and *v* and *µ* are somewhat constrained by the literature^25,33,34^, although uncertainties exist due to their scale-dependence and the highly fractured and heterolithic nature of volcanic materials. Based on the abundance of compliant breccia and pyroclastic materials in Volcán Taburiente, we have chosen to use a Young’s modulus of 2 GPa and Poisson’s ratio of 0.25, which corresponds to a shear modulus of 0.8 GPa. Viscosity is less well constrained, but is generally thought to be on the order of 10^22^–10^23^ Pas for basaltic rocks at shallow depths and low temperatures^9^. We tried a range of *µ* between 1 × 10^22^ and 5 × 10^22^ Pas (Supplementary Fig. S3). The model is insensitive to Poisson’s ratio.

The remaining variables in Eq. (1) (*P*_0_, *f*_*d*_ and *h*) can be related directly to the observed dyke aperture distribution and final bulk strain. *P*_0_ is related to the largest observed aperture using Eq. (1), and given we are using *E* = 2 GPa, our field observations (Fig. 3) suggest that it should be 60–70 MPa. All three terms can also be related directly to the observed final strain, the accommodation stress asymptote, and hence the mode of the aperture distribution (see the Supplementary Method for more details). Thus, we can estimate these terms by fitting them to our measurements of dyke aperture and spacing (see Supplementary Fig. S3).

Finally, we perturbed the overpressure at each timestep (using a numerical solution to Eq. (4)) such that it followed a normal distribution with a mean of *P*_0_ and a standard deviation that was optimised (along with *P*_0_, *f*_*d*_ and *h,* as previously described) to fit the observed aperture distribution. Overpressure was modelled as a stochastic variable to account for natural variations in the yield stress at which the source magma chamber fails during each dyking event^37^ and variations in magma buoyancy and viscosity.

**Figure 5:**
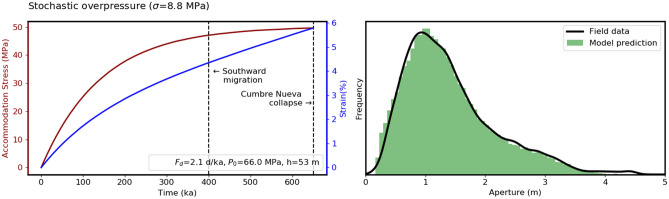
A numerical solution to the previously described Maxwell accommodation model using the elastic parameters described in the text, randomly varying magma pressure sampled from a normal distribution, and calibrated using Powell minimization to fit observed aperture data. The optimised parameters (µ = 3.0 × 10^22^, F_d_ = 2.1 days/ka, h = 53 m and P = Ν(µ = 66, σ = 8.8) MPa) give results that closely match the observations. The final bulk strain of 5.5% and equilibrium excess pressure of 17.5 MPa were not used to calibrate the model, and match field estimates (Figs. 3, 4) excellently. Note, however, that this is probably not a unique solution and so there may be other parameter combinations that reproduce the observed aperture data equally well.

The estimated apertures (Fig. 5) show a close fit to our observations, and the optimised parameters are geologically reasonable with the exception of dyke height (53 m), which is significantly less than we would expect. Using an elastic modulus of 2 GPa in Eq. (1) and excess pressures of 65–80 MPa, > 100 m high dykes would be 10 s of metres thick, which has not been observed.

This discrepancy can be explained if *h* instead represents an effective elastic height, which is significantly less than the actual height of large dykes due to (1) partial closure prior to solidification, and (2) bonding between either side of the fracture due to unbroken rock bridges (which are commonly observed on La Palma and elsewhere as ‘step-overs’; Supplementary Fig. S2). Unbroken rock-bridges tying together nominal fracture surfaces are commonly observed in laboratory experiments examining fluid-driven rock fracture; a striking example is shown in Fig. 24 of Hampton et al.^38^. However, the quantitative impact of these rock-bridges on fracture aperture remains an unresolved topic of ongoing research.

## Discussion

Because petrological constraints indicate magma is not stored at shallow depths below La Palma^20,21^, we suggest the Taburiente dykes are radial because of topographic stresses rather than a shallow pressure source. The temporal progression of dyke orientations from NW striking towards radial observed from deeper parts of the edifice (Supplementary Fig. S1) is also consistent with deflection due to increasing edifice load. Early intrusions would have formed in a NW orientation because of a combination of NNW-oriented regional maximum horizontal stress, post-collapse stress modification^39^ and topography associated with the Garafia volcano. As the Taburiente edifice grew (probably quite rapidly^16^), topographic stresses progressively came to dominate regional stresses, favouring more radial dyke orientations.

Due to the topographic source of radial stress in Volcán Taburiente, dykes need not have propagated away from the dyke swarm focal point. Indeed, this is unlikely given, (1) the lack of evidence for shallow storage, and (2) the variable plunge-directions of observed flow indicators. Instead, we suggest that dykes ascending through the crust re-oriented into radial orientations as they entered the region below Volcán Taburiente where topographic stresses became dominant. In our conceptual model (Fig. 1), dykes propagate laterally as they are deflected away from regions of elevated topographic stress^40^, and possibly also due to decreasing buoyancy at shallow depths, forming blade-shaped intrusions (e.g. Supplementary Fig. S2). Similar interactions between ascending dykes and topographic loads have been suggested based on numerical models^5^ and field observations^12,25^.

The inverse correlation between dyke thickness and depth results from a shift in the balance between confining pressure (resisting fracture opening) and internal magma pressure, although a decrease in host-rock elastic modulus at shallow depths could also play a role. We suggest that the distinct range of paleo-depths (1.1–0.7 km) over which the increase in aperture occurs could be explained by the transition from supercritical to gaseous H_2_O at pressures of ~ 22 MPa^41^ (depths ~ 750–900 m). It is also plausible that less overpressured dykes are arrested or deflected before they reach the upper levels of the edifice, and hence shallower dykes tend to be thicker.

Based on the distribution of preserved eruptive centres, it has been suggested^16^ that the northern flanks of Volcán Taburiente were formed by two diffuse rift-zones trending NE and NW. Including the collapsed Cumbre Nueva ridge in the south, this would give the Taburiente edifice a ‘three-pointed-star’ type geometry that has long been proposed as typical for the Canary Islands^7^. However, based on our strain estimates (Fig. 4), we find no evidence for a concentration of dykes along the NE or NW margins of the Caldera.

Instead, our data suggests that even though dykes in the Taburiente magma plumbing system are radial, N–S orientations are favoured, causing denser dyke swarms and larger (~ 8% vs ~ 3%) extensional strain in the Hoyo Verde and Los Cantos areas. Measurements of dykes crosscutting the seamount complex also suggest a dominant N–S trend^14^. It seems plausible that this orientation was favoured from early in the growth of Volcán Taburiente due to NNW–SSE oriented regional horizontal stress^11^ and topographic loads associated with the Garafia edifice and its subsequent collapse. Abundant proximal pyroclastic deposits exposed immediately above the basal unconformity of Volcán Taburiente at Los Cantos support this hypothesis, as they suggest significant early volcanic activity in the north of the Caldera.

The location of the focal point of the radial dykes provides an additional constraint on the geometry of Volcán Taburiente, as it should lie at the centre of the topographic stress field and hence below the edifice paleo-summit. Its position in the south of Caldera Taburiente suggests a more elongate edifice than is indicated by modern-day topography. We therefore conclude that our dyke orientation measurements and the strain induced by their emplacement are best explained by a somewhat elongated Taburiente edifice. This topography would further encourage the emplacement of N–S dykes^4^, helping to explain the strain localisation observed in the north of Caldera Taburiente and the late-stage growth of the N–S oriented Cumbre Nueva ridge.

Combining these interpretations of Volcán Taburiente’s shallow magma plumbing system with our Maxwell visco-elastic model for radial dyke accommodation, we propose that a combination of topographic and remote tectonic stresses governed dyke-propagation paths, while dyke induced strain was accommodated visco-elastically. Accommodation stresses associated with this visco-elastic deformation will have caused stress to evolve towards an isotropic state within heavily intruded portions of the edifice. Subsequent dykes would have been deflected away from these regions of high stress^6,36^, causing volcanic activity to migrate. In our model (Fig. 5) the accommodation stress reaches a maximum at about the same time that eruptive activity began to focus on the southern flank of Volcán Taburiente, forming the Cumbre Nueva ridge. We propose that this shift in activity occurred as dykes were deflected southwards by a ‘stress-plug’ formed near the centre of the radial dyke swarm (below the summit of Volcán Taburiente), noting that escape to the north would have been blocked by stresses related to the older Garafia edifice^16^.

The transition from radial to focused dyking below the Cumbre Nueva ridge can thus be explained by accommodation stresses blocking dyke ascent below the summit of Volcán Taburiente and regional tectonic stress favouring N–S oriented dykes. If widespread, this process of stress-plug development followed by lateral escape may help to explain the apparent self-organisation of many volcanic systems into elongate volcanic ridges or rift zones. The competition between stress plug development due to intrusive activity and counteracting topographic stress changes due to eruptive activity and/or edifice instability could also be a significant control on the evolution of magma plumbing systems and distribution of associated volcanism.

The close fit between field measurements and the apertures predicted by our Maxwell visco-elastic model highlight the influence of accommodation stress on dyke apertures. Volcanic systems typically have long-tailed dyke aperture distributions, which have previously been interpreted to reflect the distribution of magma chamber failure pressures^33^. Our results show that this need not be the case. Instead, the build-up of accommodation stress can also cause long-tailed dyke aperture distributions, regardless of the distribution of source overpressures. These effects must be considered should aperture data be used to estimate source properties^33,37,42^, as these methods typically assume lithostatic stress.

We conclude that the stresses induced by successive intrusions accumulate in volcanic edifices and influence dyke propagation paths and apertures. The stress field within heavily intruded regions may be significantly different to that predicted by a lithostatic model, because dyke-induced deformation increases horizontal stress magnitude and in doing so reduces the differential stress. Stress plug development by this mechanism may be an important and widespread control on the spatio-temporal distribution of volcanism in long-lived volcanic systems.

## Methods

### Digital mapping

To circumvent limited access to steep and unstable exposures within Caldera Taburiente, 14 unmanned aerial vehicle (UAV) surveys were conducted over ~ 2–50 hectare areas. Survey sites were chosen to capture a representative distribution of the available exposure within Caldera Taburiente and a range of different depths. Imagery was collected using a DJI Phantom 4 Pro and its integrated camera (20–megapixel CMOS sensor) along horizontal flight lines ~ 30–60 m from the cliff faces using horizontal and ~ 30 degree downward oriented viewing angles and vertical and horizontal overlaps of ~ 80%. These image sets were then reconstructed using a structure-from-motion multi-view-stereo (SfM-MVS) workflow^30,43^ to create a database of 3-D digital outcrop point cloud models at ground sampling distances of ~ 2–5 cm. Specific details of each of the surveys is included in the Supplementary Method and the dataset can be downloaded from https://doi.org/10.26180/5d688c17f2ed2.

The upper and lower surfaces of 519 intrusions within these models were then digitised from the high-resolution 3-D point clouds using the Compass plugin in Cloud Compare^31^. Continuous exposure within the survey areas means that all intrusions > 20 cm thick could be identified, allowing the number and spacing of intrusions in each survey area to be characterised. Structure-normal estimates (SNEs)^30^ constraining dyke orientation and true thickness were also generated at each point along the intrusion contacts. These were manually vetted to remove invalid results in non-planar sections of the dyke or where data quality was poor^30^, after which 12 million SNEs from ~ 400,000 locations remained, covering ~ 60% of the 66 km of digitised dyke margins.

### Strain estimation

Dykes in each survey were divided into horizontal scan-lines spaced at intervals of 1 m vertically to investigate the bulk-strain associated with their emplacement. Assuming Mode I opening, the dilation vector of each dyke along a scan line was calculated from the SNEs by multiplying the structure normal vector by the dyke thickness. These opening vectors were summed along each scan line, and the resultant expressed in radial and tangential components based on the maximum likelihood radial centre. The same method was used to estimate vertical strain at each location, although in this case vertical scan lines were used and both dykes and inclined sheets were included.

### Visco-elastic modelling

Python code for our visco-elastic modelling and the generation of Supplementary Fig. S3 and Fig. 5 is included in the Supplementary Method, along with the derivation of our Maxwell constitutive equation (Eq. (2)).

## Supplementary information

Supplementary Information.

## Data Availability

The digital outcrop models and associated structural measurements analysed during this study are available on the FigShare repository, https://doi.org/10.26180/5d688c17f2ed2. The python code used to perform our analyses is included in the Supplementary Information files.
