# Supplementary material for: Dyke apertures record stress accumulation during sustained volcanism
Source: Sci Rep. 2020 Oct 15;10:17335. doi: 10.1038/s41598-020-74361-w (PMC7566645; doi:10.1038/s41598-020-74361-w)

Supplementary Material for

Dyke apertures record stress accumulation during sustained volcanism

Samuel T. Thiele[1,2], Alexander R. Cruden[1], Steven Micklethwaite[1], Andrew P. Bunger[3,4], Jonas Köpping[1]

[1]*School of Earth, Atmosphere and Environment, Monash University, Melbourne, 3800, Australia*
[2]*Helmholtz Institute Freiberg for Resource Technology, Helmholtz-Zentrum Dresden-Rossendorf, 09599 Freiberg, Germany*
[3]*Department of Civil and Environmental Engineering, University of Pittsburgh, Pittsburgh, PA, 15269, USA*
[4]*Department of Chemical and Petroleum Engineering, University of Pittsburgh, Pittsburgh, PA, 15269, USA*

SUPPLEMENTARY FIGURES

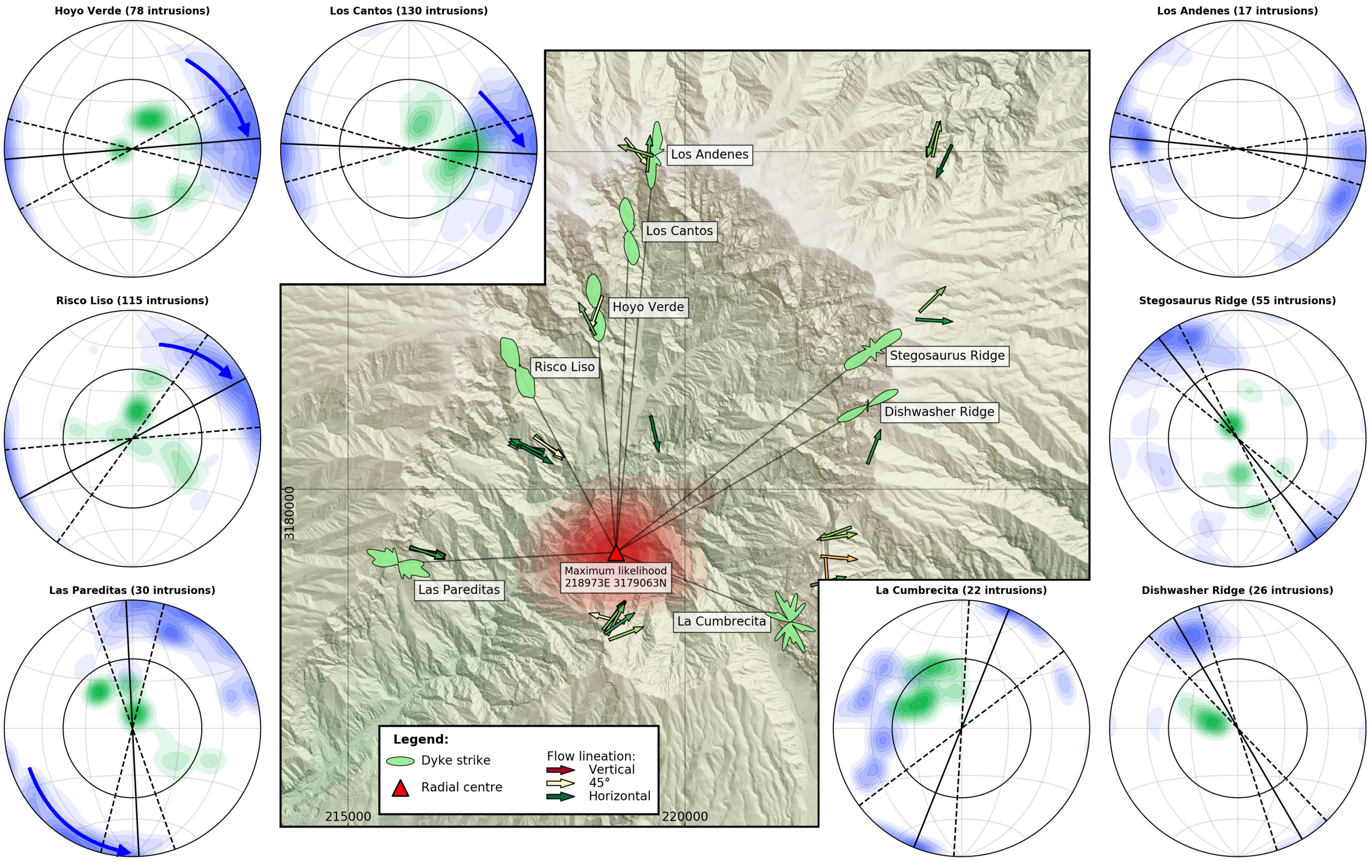

Figure S1. Structural data extracted from the UAV surveys and used to constrain the focal point of the radial dyke swarm. Density contours of poles to dykes dipping $> 45°$(blue) and shallow-dipping ($< 45°$) inclined sheets and sills (green) are plotted for each survey area on lower-hemisphere stereographic projections (a.k.a., stereonets). The maximum likelihood radial trend is plotted on each stereonet (black solid line) and 90% credible intervals shown as black dashed lines. Dyke strikes are also plotted as pale green polar kernel density estimates (rose diagrams) on the map at each survey location. The maximum likelihood swarm centre (red triangle) is also shown, and associated uncertainty highlighted by plotting contours of the log likelihood function in red. Flow lineations defined by field indicators such as stretched vesicles and striated margins are also plotted as arrows pointing in the down-dip direction and coloured according to the lineation's plunge, highlighting the variable but generally sub-horizontal to shallowly-inclined magma flow.

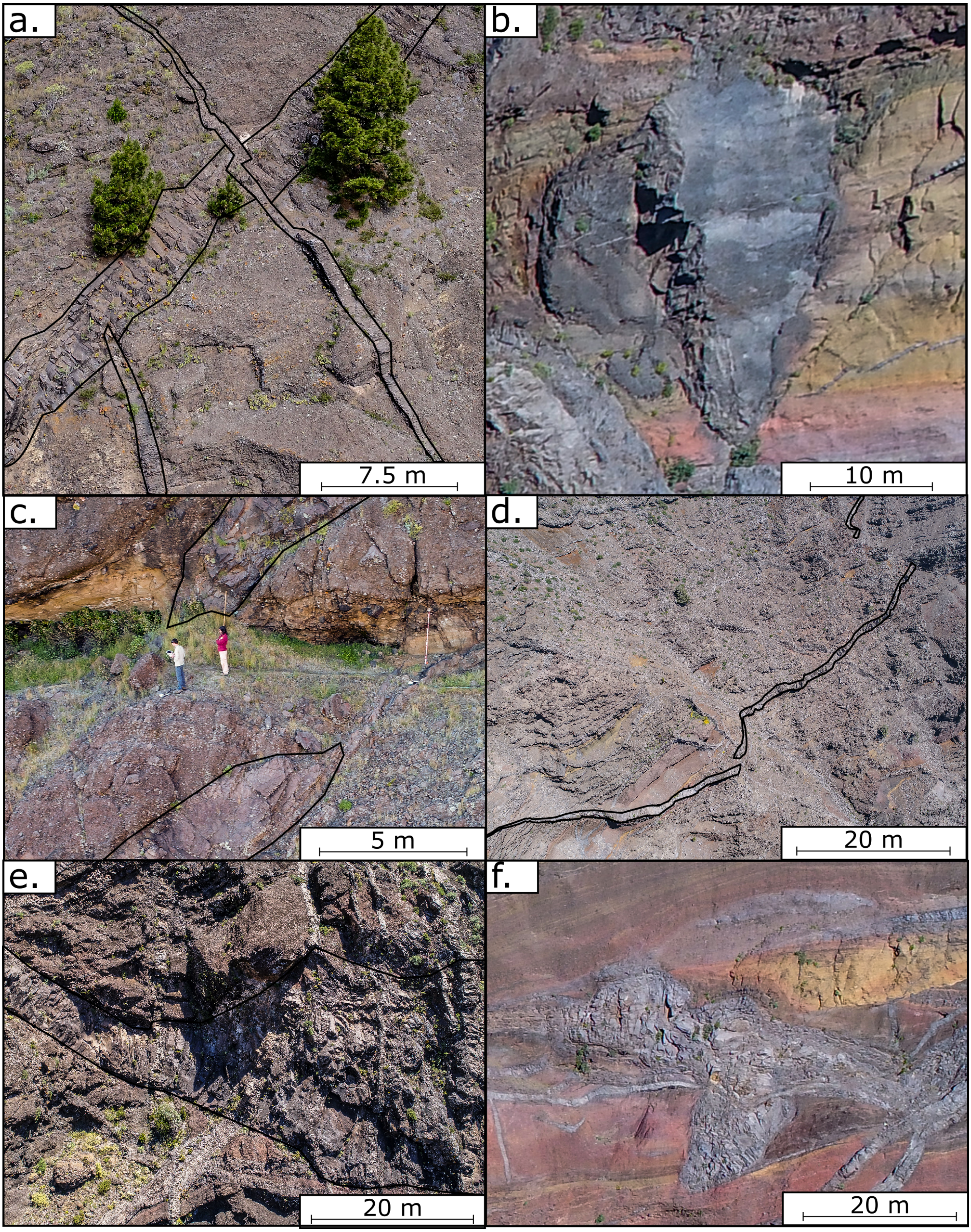

Figure S2. Field images of intrusions exposed in cliffs within Caldera Taburiente including: (a) an inclined sheet crosscut by thin radial dykes; (b) blade-shaped dyke in phreatomagmatic tuff; (c) Dyke step-over; (d) saucer-shaped sill; (e) variably thick sill crosscutting scoria; (f) irregular intrusion in altered pyroclastic deposits.

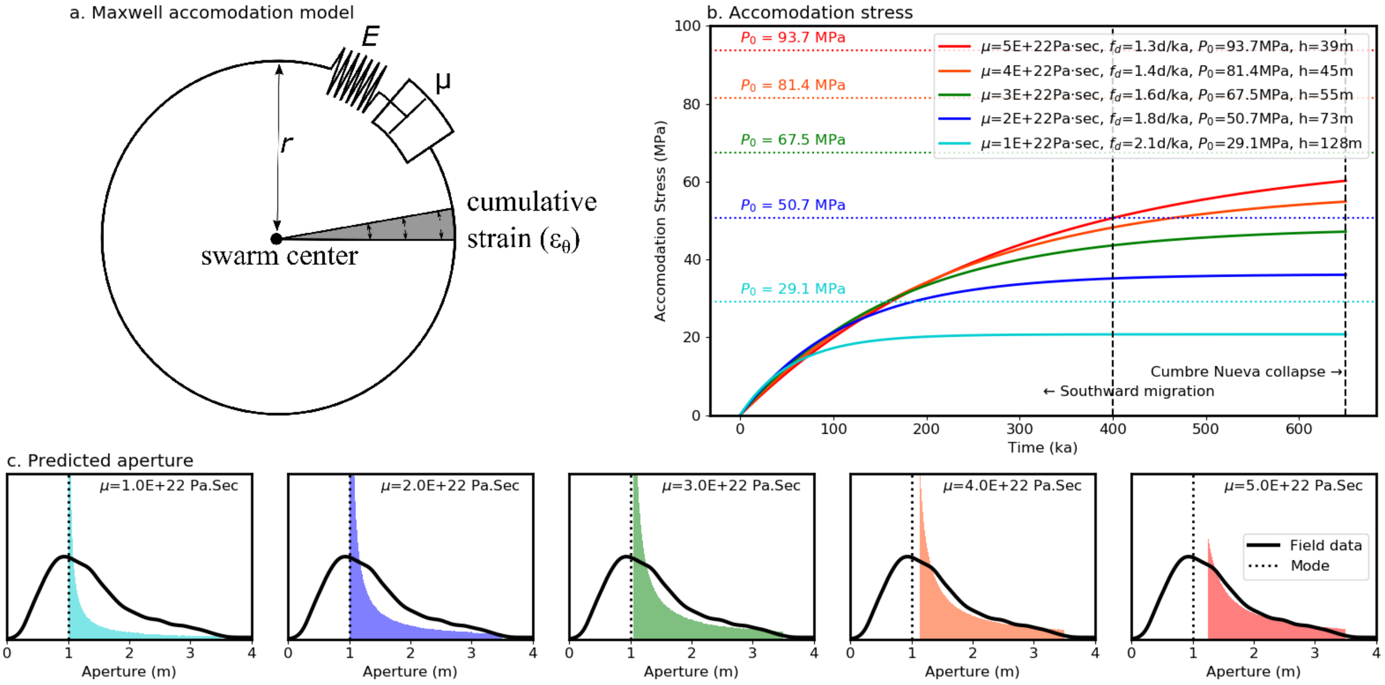

Figure S3. Stress accumulation assuming a Maxwell viscoelastic rheology for a volcanic edifice with Young's modulus of 2 GPa, Poisson's ratio of 0.25 and viscosity (μ) of 1-5$\times10^{22}$ Pas. The constant rate of dyke-injection (F_d), dyke height h and initial excess pressure P_0 are determined from observed maximum and mode dyke apertures (which relate to the initial and equilibrium excess pressure) and estimates of the bulk tangential strain using a least-squares solver. The results for viscosities of 3-4$\times10^{22}$ Pas match observed dyke aperture distributions and reach an equilibrium state after 400 ka, which corresponds to the amount of time it took for activity to localise onto the southern flank of Volcan Taburiente.

SUPPLEMENTARY METHODS

Photogrammetric survey methodology

Seventeen UAV surveys were conducted in May 2017 with approval and assistance from Parque National Caldera de Taburiente, using a DJI Phantom 4 Pro and its integrated 20-megapixel camera. Survey methodology was tailored to the topography being captured: cliff faces were flown manually using horizontal flight lines and horizontal + 30 degree downward oriented viewing angles, while MapPilot was used to survey flatter areas, using a nadir viewing angle and overlaps of 80% along flight lines and 70% between them. The target ground sampling distance (GSD) was a compromise between desired survey area and the features of interest, and so varies between surveys (Table 1). Generally, large cliff-exposures (e.g. Risco Liso) were flown at a distance of >50 m to enable a large survey area (at lower GSD), while smaller areas were flown at distances of 10-50 m to resolve smaller features.

Poor GPS reception and limited or impossible access to survey areas precluded the employment of accurately surveyed ground control points in most cases (Table 1). Instead, the surveys were approximately georeferenced using on-board GPS data and then aligned to the publically available 2014 Spanish LiDAR survey series (which has a resolution of 0.5 to 0.75 points/m2) after photogrammetric reconstruction. This alignment was done using the iterative closest point (ICP) algorithm in CloudCompare. All of the surveys were co-registered to this LiDAR data to within 1-2 m, suggesting no systematic errors or distortions in the final digital outcrop models. Structure-from-motion multi-view-stereo (SfM-MVS) photogrammetric reconstruction was performed using Agisoft Photoscan Professional (now called Agisoft Metashape) version 1.4.3. Image contrast and colour balance was enhanced using a batch-script in Adobe Photoshop (such that the same operation was applied to all images in a survey) prior to reconstruction.

Table S1: UAV photogrammetry surveys and associated survey method (either manual, or using both MapPilot and manually taken images) and other parameters.

Survey name	Method	Image count	GSD (cm/px)	Georeferencing method	Area (km2)	Sparse cloud (points)	Dense cloud (million points)
Las Pareditas 1	manual	49	2.5	Direct+ICP	0.0158	28,103	11
Las Pareditas 2	manual	95	3.2	Direct+ICP	0.0911	43,427	32
Las Pareditas 3	manual	148	2.7	Direct+ICP	0.0674	44,649	151
Risco Liso cliff	manual	361	5.0	Direct+ICP	0.517	168,602	66
Risco Liso summit	both	64	6.5	Direct+ICP	0.27	55,991	112
Hoyo Verde Alto	manual	201	1.0	DGPS+Direct+ICP	0.0419	177,156	100
Hoyo Verde Cliff	manual	196	1.8	Direct+ICP	0.0593	97,758	64
Hoyo Verde Rio	both	251	0.001	Scalebars+ICP	1220 m^2	196,035	115
Los Cantos 1	manual	108	6	Direct+ICP	0.176	37,792	20
Los Cantos 2	manual	258	5.7	Direct+ICP	0.345	88,904	59
Los Andenes norte	both	512	1.3	DGPS+Direct+ICP	0.048	294,482	209
Los Andes sur	both	215	2.0	DGPS+Direct+ICP	0.078	129,533	77
Stegosaurus Ridge	both	249	5	Direct+ICP	0.158	69,389	42
Dishwasher Ridge	manual	77	6.5	Direct+ICP	0.328	33,569	32
La Cumbrecita	manual	211	5.0	Direct+ICP	0.293	71,088	47
Bejenado Lava Worm	manual	162	2.63	Direct+ICP	0.038	56,345	32
Socomo	both	148	3.5	Direct	0.358	117,983	77

Maxwell model derivation

If radial dykes are emplaced into a volcanic edifice that deforms visco-elastically, the tangential (hoop) stress that accumulates during repeated dyke emplacement can be investigated using a 1D Maxwell visco-elastic model. Here we detail the assumptions and derivation of the model beginning with a generalized three-dimensional viscoelastic model.

Assumptions and boundary conditions

First, we assume that:

(1) Ascending dykes are deflected towards areas of low stress, such that long-term averaged circumferential stress σ_θ is spatially uniform (at least to some critical distance). This assumption means that the intrusions induce a change in the circumferential normal strain ϵ_θ that is spatially constant.

(2) Dyke intrusion does not change vertical stress (due to the free surface). Adopting a notation where σ and ϵ are taken as changes in stress and strain, respectively, due to the dyke intrusion, this assumption is stated as $\sigma_z = 0$.

(3) The edifice is free to move in the r direction (due to its conical shape), meaning $\sigma_r = 0$.

(4) The r, θ, z directions define the principal axes for the induced stresses and strains, that is, there are no induced shear stresses or strains in this coordinate system.

From these assumption, we can express the change in the stress tensor as:

$$\bar{\sigma} = \begin{bmatrix} 0 & 0 & 0 \\ 0 & \sigma_\theta & 0 \\ 0 & 0 & 0 \end{bmatrix} . \tag{1}$$

Using Hooke's law, a purely elastic response to this stress would be:

$$\bar{\epsilon} = \begin{bmatrix} \frac{-v}{E}\sigma_\theta & 0 & 0 \\ 0 & \frac{1}{E}\sigma_\theta & 0 \\ 0 & 0 & \frac{-v}{E}\sigma_\theta \end{bmatrix} , \tag{2}$$

where E and ν are the Young's modulus and Poisson's ratio, respectively. It is often convenient in constitutive modeling to separate stress and strain into their volumetric and deviatoric parts. So, by decomposition we can write that:

$$\sigma_{ij} = s_{ij} + \frac{1}{3}\delta_{ij}\hat{\sigma}_{kk} \implies \bar{\sigma} = \begin{bmatrix} -\frac{1}{3}\sigma_\theta & 0 & 0 \\ 0 & \frac{2}{3}\sigma_\theta & 0 \\ 0 & 0 & -\frac{1}{3}\sigma_\theta \end{bmatrix} + \begin{bmatrix} \frac{1}{3}\sigma_\theta & 0 & 0 \\ 0 & \frac{1}{3}\sigma_\theta & 0 \\ 0 & 0 & \frac{1}{3}\sigma_\theta \end{bmatrix} , \tag{3}$$

$$\epsilon_{ij} = e_{ij} + \frac{1}{3}\delta_{ij}\hat{\epsilon}_{kk} \implies \bar{\epsilon} = \begin{bmatrix} -\frac{1+v}{3E}\sigma_\theta & 0 & 0 \\ 0 & \frac{2(1+v)}{3E}\sigma_\theta & 0 \\ 0 & 0 & -\frac{1(1+v)}{3E}\sigma_\theta \end{bmatrix} + \begin{bmatrix} \frac{1-2v}{3E}\sigma_\theta & 0 & 0 \\ 0 & \frac{1-2v}{3E}\sigma_\theta & 0 \\ 0 & 0 & \frac{1-2v}{3E}\sigma_\theta \end{bmatrix} , \tag{4}$$

where δ_{ij} is the Kronecker delta and s_{ij} and e_{ij} are the deviatoric components of the stress and strain tensors.

3D visco-elasticity:

A general form of a 3D viscoelastic behaviour is then given by (e.g. [1]):

$$\{P\}s_{ij} = 2\{Q\}e_{ij} , \tag{5}$$

$$\{M\}\sigma_{ii} = 3\{N\}\hat{\epsilon}_{ii} , \tag{6}$$

where the { } notation indicates differential operators of the form

$$\{P\} = \sum_{i=0}^{m} P_i \frac{\partial^i}{\partial t^i} . \tag{7}$$

Expanding this for first-order (Maxwell) viscoelasticity ($m = 1$), and accounting for the uniaxial stress and zero shear stresses/strains implied by the assumptions outlined above gives, and considering the circumferential normal stress strain relationship ($i, j = 2, 2$) from the deviatoric part of the constitutive law, leads to

$$P_0\sigma_\theta + P_1\dot{\sigma}_\theta = 3Q_0 e_\theta + 3Q_1\dot{e}_\theta \ . \tag{8}$$

Rearranging Eq. 8. gives

$$\dot{e}_\theta = \frac{P_0}{3Q_1}\sigma_\theta + \frac{P_1}{3Q_1}\dot{\sigma}_\theta - \frac{Q_0}{Q_1}e_\theta \ . \tag{9}$$

Next we will prescribe the parameters of the constitutive law in order to recover Hooke's law under rapid deformation and viscous relaxation under slow deformation. The material does not relax due to instantaneous strain, which causes a purely elastic response, so from Hooke's law (Eq. 4):

$$e_\theta = \frac{2(1+v)}{3E}\sigma_\theta \Rightarrow \dot{e}_\theta = \frac{2(1+v)}{3E}\dot{\sigma}_\theta \ , \tag{10}$$

$$\frac{P_1}{3Q_1} = \frac{2(1+v)}{3E} = \frac{1}{3G} \ . \tag{11}$$

Here G is the shear modulus. Similarly, if the strain rate is very slow then strain will be entirely viscous and it will not induce a change in stress, so

$$\dot{e}_\theta = \frac{P_0}{3Q_1}\sigma_\theta - \frac{Q_0}{Q_1}e_\theta \ , \tag{12}$$

$$\sigma_\theta = \frac{3Q_1}{P_0}\dot{e}_\theta + \frac{3Q_0}{P_0}e_\theta \ . \tag{13}$$

When viewed in this way it is evident that e_θ is an initial value before the strain starts slowyly changing. Hence, as we are interestied in solving for stress-changes relative to an initial value before the intrusions form, we can use $e_\theta = 0$. Similarly, we can use μ to describe the viscosity of the rock (under uniaxial strain), such that:

$$\sigma_\theta = \mu\dot{e}_\theta, \qquad e_\theta = \epsilon_\theta - \frac{\epsilon_r + \epsilon_\theta + \epsilon_z}{3} \ , \tag{14}$$

$$\frac{3Q_1}{P_0} = \frac{3\mu}{2} \ . \tag{15}$$

Substituting into the generalized constitutive law leads to a constitutive equation for the material under uniaxial circumferential stress given by

$$\dot{e}_\theta = \frac{2\sigma_\theta}{3\mu} + \frac{\dot{\sigma}_\theta}{3G} \ . \tag{16}$$

Hence, if $\dot{\Delta}$ is the strain rate induced by the emplacement of the dykes, then

$$\dot{\epsilon} = \begin{bmatrix} 0 & 0 & 0 \\ 0 & \dot{\Delta} & 0 \\ 0 & 0 & 0 \end{bmatrix} \ . \tag{17}$$

Thus we can relate the stress induced by the intrusions to the rate at which they induce strain using the 1D Maxwell-type viscoelastic equation

$$\dot{\Delta} = \frac{\sigma_\theta}{\mu} + \frac{\dot{\sigma}_\theta}{2G} \ . \tag{18}$$

Relationship to overpressure

In our instance, the induced strain rate $\dot{\Delta}$ will depend on two factors: the rate of dyke injections F_d and the aperture a of those dykes. Assuming the dykes are propagating laterally (as suggested by field observations) and have longer strike-length than height h, their aperture can be linked to the stress when they form:

$$a = \frac{2h(1-v^2)}{E}(P_0 - \sigma_\theta), \text{ providing } P_0 > \sigma_\theta \ . \tag{19}$$

If we assume a constant rate of dyke injections F_d along a circumference with length $L = 2\pi r$, strain rate becomes:

$$\dot{\Delta} = F_d \frac{a}{L} = F_d \frac{2h(1-v^2)}{E \times L}(P_0 - \sigma_\theta) \ . \tag{20}$$

Hence, as per the constitutive relationship derived above:

$$\frac{1}{2G}\dot{\sigma_\theta} + \frac{1}{\mu}\sigma_\theta = k(P_0 - \sigma_\theta), \tag{21}$$

where, for convenience:

$$k = F_d \frac{2h(1-v^2)}{E \times L} \ . \tag{22}$$

This expands to give a linear differential equation in standard form:

$$\dot{\sigma_\theta} + 2G(\frac{1}{\mu} + k)\sigma_\theta = 2kGP_0 \ , \tag{23}$$

which can be solved analytically to give stress as a function of time assuming $\sigma_{t=0} = 0$:

$$\sigma_\theta(t) = k\Big(\frac{P_0}{\frac{1}{\mu}+k} - \frac{P_0}{\frac{1}{\mu}+k}e^{-2G(\frac{1}{\mu}+k)t}\Big) \ . \tag{24}$$

[1] G.E. Mase. *Schaum's Outline Of Theory And Problems Of Continuum Mechanics*. McGraw-Hill Book Co., 1970.

ANALYSIS AND IMPLEMENTATION

All computations for this paper, including analysis of the structural data and computation of the Maxwell models, were implemented in Python notebooks. These have been included below (1) for reference and (2) to highlight how the pycompass package can be used to analyse huge structural datasets extracted from digital outcrop models.

Structural analyses

Dyke orientation and thickness measurements extracted from the UAV surveys using the Compass plugin in CloudCompare have been exported to a .xml format that can be loaded and analysed using pycompass. These .xml files are avaliable here.

The following notebook provides a complete record of this workflow and the logic behind our structural analyses. Specifically, it contains the code required to create Figures 2, 3, 4, and S1.

Structural Analysis Notebook

```
In [129]: import sys, os, glob
          import numpy as np

          #stats functions
          import scipy.stats
          import wquantiles
          import pandas
          from sklearn.neighbors import KernelDensity
          from collections import OrderedDict
          from scipy.stats import gaussian_kde

          #plotting functions
          import matplotlib as mpl
          import matplotlib.pyplot as plt
          import matplotlib.cm as cm
          from matplotlib.colors import LightSource
          from matplotlib.gridspec import GridSpec
          import mplstereonet
          import colorsys
          from matplotlib.patches import Arc

          #gis
          import georasters
          import utm

          #load pyCompass classes and tools
          sys.path.append("../libs")
          from pycompass.IOTools import ccXML
          from pycompass.SNE import pdf
          from pycompass.SNE import SNEList
          from pycompass.SNE import CombinedSNEList

          #ipython commands
          from IPython.display import clear_output
          %matplotlib inline
```

1 Load data

Intrusion traces, orientation estimates, thicknesses measurements etc. have been exported into an .xml format for analysis. For convenience, we load all these datasets and group them into classes that make access convenient. During this step we also classify the intrusions into a steeply dipping (dyke) and shallow-dipping (sheet-intrusion) set.

```
In [135]: #load data files
          dataDir = "../data"

          #data file paths
          #,
          path = [ "las_pareditas_2.xml", "risco_liso_summit.xml","risco_liso_cliff.xml",
                  "hoyo_verde_alto.xml", "hoyo_verde_cliff.xml", "los_cantos_1.xml","los_cantos_2.xml",
                  "stegosaurus_ridge.xml","dishwasher_ridge.xml","la_cumbrecita.xml",
                  "los_andenes_sur.xml","las_pareditas_1.xml","las_pareditas_3.xml","los_andenes_norte.xml"]

          #some datasets are merged (later on)
          groups = [ [0,11,12], #las pareditas
                     [1,2], #risco liso
                     [3,4], #hoyo verde
                     [5,6], #los cantos
                     [7], #stegosaurus ridge
                     [8], #dishwasher ridge
                     [9], #la cumbrecita
```

```
             [10,13]]#los andenes

    #names of the groups
    #"Los Andenes",
    titles = [ "Las Pareditas", "Risco Liso","Hoyo Verde", "Los Cantos",
              "Stegosaurus Ridge","Dishwasher Ridge","La Cumbrecita","Los Andenes"]

    data = [ccXML( os.path.join(dataDir,f) ) for f in path]
```

In [136]:
```
#utility class for storing dyke surfaces and SNEs (and slicing this data based on elevation)
class Dyke:
    def __init__(self,name,upper,lower,SNE):
        self.name = name
        self.upper = np.array(upper)
        self.lower = np.array(lower)
        self.SNE = SNE

    def slice(self,minz,maxz):
        umask = np.logical_and(self.upper[2] > minz,self.upper[2] < maxz)
        lmask = np.logical_and(self.lower[2] > minz,self.lower[2] < maxz)

        sx = []
        sy = []
        sz = []
        if np.any(umask):
            sx = np.append(sx,self.upper[0][umask])
            sy = np.append(sy,self.upper[1][umask])
            sz = np.append(sz,self.upper[2][umask])
        if np.any(lmask):
            sx = np.append(sx,self.lower[0][lmask])
            sy = np.append(sy,self.lower[1][lmask])
            sz = np.append(sz,self.lower[2][lmask])

        if len(sx) == 0: #dyke not in slice
            return None,None,None,None

        #compute centroid
        centroid = [np.mean(sx),np.mean(sy),np.mean(sz)]

        #slice SNEs
        smask = np.logical_and(self.SNE.pos[2] > minz,self.SNE.pos[2] < maxz)
        thick = self.SNE.thickness #by default use all SNEs
        trend = self.SNE.trend
        plunge = self.SNE.plunge
        if np.any(smask): #if any SNEs are inside slice, use only local ones
            thick = self.SNE.thickness[smask]
            trend = self.SNE.trend[smask]
            plunge = self.SNE.plunge[smask]

        #compute mean orientation and thickness
        mthick = np.mean(thick)
        vsum = np.zeros(3)
        iv = pdf.trendPlunge2Vec( np.deg2rad(trend[0]), np.deg2rad(plunge[0]) )
        for i in range(len(trend)):
            v = pdf.trendPlunge2Vec(np.deg2rad(trend[i]),np.deg2rad(plunge[i]))
            if np.dot(v,iv) > 0:
                vsum += v
            else:
                vsum -= v
        vsum /= len(trend)
        mtrend,mplunge = np.rad2deg(pdf.vec2TrendPlunge(vsum))

        return centroid,mtrend,mplunge,mthick
```

In [137]:
```
#extract sheet intrusions and classify into steep & shallow dipping sets.
def extractIntrusions(d,minSteep=45):
    steep = []
    shallow = []

    #loop through geo-objects in file
    geoObjects = d.filterByKey("GEO_OBJECT")
    for g in geoObjects:
        #ignore non-intrusions
```

```
        if not ("dyke" in g['@name'].lower()) or ("sill" in g['@name'].lower()) or ("intrusion" in g['@name'].lower()):
            continue

        #get SNE object
        S = SNEList.loadFromData(d,g)

        if len(S.trend) < 10:
            print("Ignoring dyke %s as it has insufficient (or no) SNEs." % g['@name'])
            continue

        #gather traces from upper and lower surface of dyke
        names,upper,interior,lower = d.extractRegions(data=g)
        upper = d.extractTraces(data=upper[0])
        lower = d.extractTraces(data=lower[0])

        #merge traces
        up = np.array([[],[],[],[],[],[]])
        for t in upper:
            up = np.append(up,t,axis=1)

        lp =np.array([[],[],[],[],[],[]])
        for l in lower:
            lp = np.append(lp,l,axis=1)

        #build dyke object
        dyke = Dyke(g['@name'].lower(),up,lp,S)

        #steep or shallow?
        p = S.evalP1D("dip",45,90)
        if p < 0.5: #dyke is more likely to be shallow dipping
            shallow.append(dyke)
        else: #dyke is steep dipping
            steep.append(dyke)

    #return
    return steep, shallow
```

```
In [138]: #lists of intrusions
          shallow = []
          steep = []
          for ids in groups:

              #loop through datasets and gather
              st = []
              sh = []
              for i in ids:
                  _st,_sh = extractIntrusions( data[i] )
                  st += _st
                  sh += _sh

              #store all dykes associated with this site
              shallow.append(sh)
              steep.append(st)

          clear_output()
```

2 Aggregate SNEs

Some of the surveys are very close together, so for convenience we lump them together to give the 8 aggregated datasets shown in Fig. 2.

```
In [139]: agg_steep = []
          agg_shallow = []
          for i in range(len(groups)): #loop through field sites
              #create combined SNE objects
              if len(steep[i]) > 0:
                  agg_steep.append( CombinedSNEList(titles[i],
                                    [d.SNE for d in steep[i]] ) )
              else:
                  agg_steep.append(None)
```

```
        if len(shallow[i]) > 0:
            agg_shallow.append( CombinedSNEList(titles[i],
                                  [d.SNE for d in shallow[i]] ) )
        else:
            agg_shallow.append(None)
```

3 Orientation

3.1 Estimate swarm centre

Many of the dykes in the above dataset are radial to an unknown swarm centre. We can constrain how well candidate swarm centres explain the observed SNEs by grid-searching the model space and evaluating a likelihood function for each candidate centre location.

First, we define our model searchspace. As the radial model has only two parameters (the x and y coordinate of the swarm center) then we can simply grid-search this whole space!

```
In [140]: #define a grid of radial centers to evaluate the likelihood for
          minx = -2500
          maxx = 5000
          miny = -5000
          maxy = 2500
          res = 50 #50 meter spacing
          _x = np.linspace(minx,maxx,int((maxx-minx)/res))
          _y = np.linspace(miny,maxy,int((maxy-miny)/res))
          xv,yv = np.meshgrid( _x, _y )

          #calculate central location of each model based on steep dykes
          centers = []
          for model in steep:
              sx = 0
              sy = 0
              n = 0
              for d in model:
                  sx += np.sum(d.lower[0]) + np.sum(d.upper[0])
                  sy += np.sum(d.lower[1]) + np.sum(d.upper[1])
                  n += len(d.lower[0]) + len(d.upper[0])
              centers.append( np.array([sx / n, sy / n]) )
```

And now evaluate the likelihood of each candidate model center based on the data kdes calculated previously:

```
In [141]: loglik = np.zeros(xv.shape)
          kappa = 15
          for i in range(xv.shape[0]):
              for j in range(xv.shape[1]):

                  #get model center
                  c = np.array([xv[i,j],yv[i,j]])

                  #evaluate against data
                  for n in range(len(centers)):

                      #calculate predicted strike (direction of radial vector from center)
                      r = centers[n] - c
                      pred = np.rad2deg( np.arctan2(r[0],r[1]) )
                      while pred < 0:
                          pred += 180
                      while pred > 180:
                          pred -= 180

                      #get likelihood for this model
                      grid,lik = agg_steep[n].getKDE("strike")
                      vm = scipy.stats.vonmises.pdf(np.deg2rad(grid),kappa,np.deg2rad(pred),0.5)
                      vm /= np.trapz(vm,grid) #normalise
                      lik *= vm
                      lik = np.trapz(lik,grid)
```

```
                    #accumulate log likelihood
                    loglik[i,j] += np.log(lik)
```

```
In [142]: #calculate maximum likelihood location
          maxidx = np.argmax(loglik)
          mlx = xv.flatten()[maxidx]
          mly = yv.flatten()[maxidx]
```

```
In [ ]: #calculate credible intervals
        CI = []
        for n,agg in enumerate(agg_steep):
            s = [] #predicted strike angle
            w = [] #weight (aka likelihood)
            for i in range(xv.shape[0]):
                for j in range(xv.shape[1]):

                    #get model center
                    c = np.array([xv[i,j],yv[i,j]])

                    #calculate predicted strike (direction of radial vector from center)
                    r = centers[n] - c
                    pred = np.rad2deg( np.arctan2(r[0],r[1]) )
                    while pred < 0:
                        pred += 180
                    while pred > 180:
                        pred -= 180

                    #store predicted strike
                    s.append(pred)

                    #get weight and append
                    w.append(np.exp(loglik[i,j]))

            r = centers[n] - np.array([mlx,mly])
            pred = np.rad2deg( np.arctan2(r[0],r[1]) ) #maximum likelihood predicted strike
            while pred < 0:
                pred += 180
            while pred > 180:
                pred -= 180

            #map predicted strike to have positive x-value (necessary for following code)
            if r[0] < 0:
                r *= -1

            #normalise predicted strikes to deviation from maximum likelihood -> avoids wrapping issues at 180 degrees...
            r = r / np.linalg.norm(r) #normalise maximum likelihood strike vector
            sv = np.array([np.sin(np.deg2rad(s)),np.cos(np.deg2rad(s))]) #convert modelled strikes to vectors
            dot = r[0]*sv[0] + r[1]*sv[1] #compute dot product between maximum likelihood strike and modelled strike
            xp = r[1]*sv[0]-r[0]*sv[1] #calculate (2D) cross product of predicted and maximum likelihood strike vector
            xp[dot < 0] *= -1
            dot = np.abs(dot)
            dev = np.rad2deg(np.arccos( dot )) #calculate acute angle between strike and max likelihood strike
            dev *= xp / np.abs(xp) #use this cross product to calulate the sign of the deviation
            dev[np.logical_not(np.isfinite(dev))] = 0 #sometimes dev is nan when pred strike == max strike

            #calculate and plot 95% confidence intervals
            l = wquantiles.quantile(dev,w,0.05) + pred
            u = wquantiles.quantile(dev,w,0.95) + pred

            while l < 0:
                l += 180
            while u < 0:
                u += 180
            while l > 180:
                l -= 180
            while u > 180:
                u -= 180

            CI.append( (l,pred,u) )
```

3.2 Plot structural data (Fig. 2)

Plots the above radial model, stereonets and rose-diagrams on a map of Caldera Taburiente.

In [144]:
```
#define projection parameters
data_origin = np.array([218000, 3182000]) #converts from data coords to UTM Z28 Coords

#convert data in local coords to UTM
centers_p = np.array(centers) + data_origin
mlx_p = mlx + data_origin[0]
mly_p = mly + data_origin[1]
```

In [145]:
```
#load topography dataset
topo = georasters.from_file("LaPalma_5m_REGC95_HU28_Merged.tif")

#calculate hillshade
ls = LightSource(azdeg=45, altdeg=45)
cmap = plt.cm.gist_earth
base = ls.shade(topo.raster,cmap=cmap, vmin=-3000,vmax=3500,
                dx=np.abs(topo.x_cell_size),dy=np.abs(topo.y_cell_size))

#reduce saturation of colormap
hsv = mpl.colors.rgb_to_hsv(base[:,:,:3])
hsv[:,:,1] *= 0.3
hsv[:,:,2] *= 0.95
base[:,:,:3] = mpl.colors.hsv_to_rgb(hsv)
```

In [146]:
```
#load flow direction vectors
df = pandas.read_csv('../data/flow_lineations.csv')

E,N,_,_ = np.array([utm.from_latlon( df["Latitude"][i], df["Longitude"][i] ) for i in range(len(df))]).T
E = np.array(E,dtype=np.float32)
N = np.array(N,dtype=np.float32)
trend = np.array(df["Trend"],dtype=np.float32)
plunge = np.array(df["Plunge"],dtype=np.float32)
```

In [147]:
```
#setup figure styles
plt.style.use(['default'])
mpl.rcParams['font.size'] = 12
mpl.rcParams['figure.titleweight'] = 'normal'
mpl.rcParams['savefig.dpi'] = 350
mpl.rcParams['axes.spines.bottom'] = True
mpl.rcParams['axes.spines.left'] = True
mpl.rcParams['axes.spines.right'] = True
mpl.rcParams['axes.spines.top'] = True
plt.rcParams["axes.edgecolor"] = "black"
plt.rcParams["axes.linewidth"] = 2
dipcmap = plt.get_cmap("RdYlGn_r")
```

In [148]:
```
#setup figure canvas
fig = plt.figure(figsize=(19,12))
gs = GridSpec(3,5)
mapAx = fig.add_subplot(gs[0:3,1:4])


dataAx = { 'Las Pareditas' : fig.add_subplot(gs[2,0],projection='stereonet'),
           'Risco Liso' : fig.add_subplot(gs[1,0],projection='stereonet'),
           "Hoyo Verde" : fig.add_subplot(gs[0,0],projection='stereonet'),
           "Los Cantos" : fig.add_subplot(gs[0,1],projection='stereonet'),
           "Los Andenes" : fig.add_subplot(gs[0,4],projection='stereonet'),
           "Stegosaurus Ridge" : fig.add_subplot(gs[1,4],projection='stereonet'),
           "Dishwasher Ridge" : fig.add_subplot(gs[2,4],projection='stereonet'),
           "La Cumbrecita" : fig.add_subplot(gs[2,3],projection='stereonet')  }

#plot topography hillshade
mapAx.imshow(base, extent=[topo.xmin,topo.xmax,topo.ymin,topo.ymax],alpha=1.0,zorder=0) #background topo

#####################################
#plot likelihood function and maxima
#####################################
#calculate normalized likelihood
lik = np.exp(loglik)
```

```python
lik /= np.max(lik)

#generate colours with 10 levels
levels = np.linspace(0,1,10)
colors = np.array([[0.8,0.1,0.1, l] for l in levels]) #min(1*np.sin(l*np.pi/2),1.0)

#plot contour
xx,yy = np.meshgrid(np.linspace(minx+data_origin[0],maxx+data_origin[0],lik.shape[0]),
                    np.linspace(miny+data_origin[1],maxy+data_origin[1],lik.shape[1]))
mapAx.contourf(xx,yy,lik,levels=levels,colors=colors)

#maximum likelihood location
mapAx.scatter(mlx_p,mly_p,color='r',marker='^',s=250,linewidths=1.5,edgecolors='k',zorder=10)
mapAx.text(mlx_p,mly_p-375,"Maximum likelihood\n%dE %dN"%(mlx_p,mly_p),
         verticalalignment='center',horizontalalignment='center',color='k',fontsize=10,
         bbox=dict(facecolor='white', alpha=0.75))

##################################################
#plot uav survey location labels
#################################################
for i,pos in enumerate(centers_p):
    if "La Cumbrecita" in titles[i]:
        mapAx.text(pos[0]-350,pos[1],titles[i], horizontalalignment='right', verticalalignment='center',color='k',
             bbox=dict(facecolor='white', alpha=0.75))
    elif "Las Pareditas" in titles[i]:
        mapAx.text(pos[0]+300,pos[1]-400,titles[i], verticalalignment='center',color='k',
             bbox=dict(facecolor='white', alpha=0.75))
    elif "Ridge" in titles[i]:
        mapAx.text(pos[0]+250,pos[1]-100,titles[i], verticalalignment='center',color='k',
             bbox=dict(facecolor='white', alpha=0.75))
    else:
        mapAx.text(pos[0]+250,pos[1],titles[i], verticalalignment='center',color='k',
             bbox=dict(facecolor='white', alpha=0.75))

#################################################
#plot rose diagrams
#################################################
for i,pos in enumerate(centers_p):
    theta,lik = agg_steep[i].getKDE("strike") #get lik function for this field site
    theta = np.deg2rad(theta) #convert to radians

    roseSize = 500 #size of rose diagram at maximum point
    scale = (lik / np.max(lik)) * roseSize #maximum likelihood

    #create polygon patch
    mapAx.fill(np.sin(theta)*scale+pos[0],np.cos(theta)*scale+pos[1],
             facecolor='lightgreen', edgecolor='k',linewidth = 0.75, alpha=0.9, zorder=5  )

    #plot radial line to this field site
    mapAx.plot([mlx_p,pos[0]],[mly_p,pos[1]],color='k',alpha=0.5)

#####################
#plot flow lineations
#####################
arrow_size = 400 #arrows are 100 m long
i = 0
for _e,_n,_t,_p in zip(E,N,trend,plunge):
    #calculate direction vector
    V = np.array([np.sin(np.deg2rad(_t)),np.cos(np.deg2rad(_t))]) * arrow_size

    #calculate start and end of arrow
    P = np.array([_e,_n]) #position of center of arrow
    start = P - V * 0.5

    #draw
    mapAx.arrow(start[0],start[1],V[0],V[1],head_width=100,
              width=40,linewidth=1.0,
              edgecolor='k',#np.array(dipcmap(_p/90))*np.array([0.5,0.5,0.5,1.0]),
              facecolor=dipcmap(_p/90),
              zorder =10)
    i += 1
```

```python
#####################
#setup map figure
####################
mapAx.set_frame_on(True)
mapAx.set_xlim(214000,226000)
mapAx.set_ylim(3175000,3186500)
mapAx.set_aspect('equal')
mapAx.set_yticks([3180000,3185000])
mapAx.set_xticks([215000,220000])
for tick in mapAx.get_yticklabels():
    tick.set_rotation('vertical')
mapAx.tick_params(direction='in',pad=-15)
mapAx.grid(color='k',alpha=0.4)

#####################
#plot stereonets
#####################
#generate colours with 10 levels in alpha
levels = np.linspace(0,1,10)
r_levels = np.array([[0.0,0.7,0.25,l] for l in levels]) #min(1*np.sin(l*np.pi/2),1.0)
o_levels = np.array([[0.3,0.4,1.0,l] for l in levels])

#plot each stereonet
for i,t in enumerate(titles):
    #get axes for this stereonet
    ax = dataAx[t]

    #get kdes
    grid, kde_steep = agg_steep[i].getKDE("ori")
    if not agg_shallow[i] is None:
        grid, kde_shallow = agg_shallow[i].getKDE("ori")

    #reshape to grid
    res = int(np.sqrt(grid.shape[1]))
    kde_steep = np.reshape(kde_steep,(res,res)).T
    if not agg_shallow[i] is None:
        kde_shallow = np.reshape(kde_shallow,(res,res)).T

    #normalise so that range is 0 - 1
    kde_steep /= np.max(kde_steep)
    kde_shallow /= np.max(kde_shallow)

    #build meshgrid of plotting coordinates (in lat,lon)
    bound = np.pi / 2
    X,Y = np.meshgrid(np.linspace(-bound,bound,res), np.linspace(-bound,bound,res)) #rectangular plot of polar data

    #plot
    cs = ax.contourf(X,Y,kde_steep,colors=o_levels,levels=levels,zorder=1)
    if not agg_shallow[i] is None:
        cs = ax.contourf(X,Y,kde_shallow,colors=r_levels,levels=levels,zorder=1)

    ax.plane(CI[i][0]+90,90, color='k',linestyle='--')
    ax.plane(CI[i][1]+90,90, color='k',linestyle='-')
    ax.plane(CI[i][2]+90,90, color='k',linestyle='--')

    #plot cone separating "steep" vs "shallow"
    ax.cone(90, 0, 45, facecolor='', zorder=4, edgecolors='k')
    ax.set_zorder(10)
    ax.set_xticks( np.deg2rad([-60,-30,0,30,60]) )
    ax.set_yticks( np.deg2rad([-60,-30,0,30,60]) )
    ax.set_longitude_grid_ends(90)
    #ax.set_azimuth_ticklabels(["","","E","","S","","W",""])
    ax.set_azimuth_ticklabels(["","","","","","","",""])
    ax.grid(color='gray',linestyle='-',alpha=0.25,lw=1.0)
    ax.set_frame_on(True)
    [i.set_linewidth(1.0) for i in ax.spines.values()]
    ax.set_title("%s (%d intrusions)" % (titles[i],len(steep[i])+len(shallow[i])), fontsize=10, fontweight='bold', y=1.0)
    ax.add_artist(mpl.patches.Rectangle((0,0),1,1,facecolor='white',fill=True,zorder=0,transform=ax.transAxes))

    #add arrows to stereonets
    start = mplstereonet.stereonet_math.line(15,70+180)
    end = mplstereonet.stereonet_math.line(3,0+180)
    dataAx["Las Pareditas"].annotate("", xy=end, xycoords='data',
```

```
                                       xytext=start, textcoords='data',
                                       arrowprops=dict(arrowstyle="simple,head_length=1.0,head_width=1.0,tail_width=0.2",
                                       connectionstyle="arc3,rad=0.3",
                                       facecolor='b',edgecolor='b'))
        start = mplstereonet.stereonet_math.line(25,15)
        end = mplstereonet.stereonet_math.line(10,60)
        dataAx["Risco Liso"].annotate("", xy=end, xycoords='data',
                                       xytext=start, textcoords='data',
                                       arrowprops=dict(arrowstyle="simple,head_length=1.0,head_width=1.0,tail_width=0.2",
                                       connectionstyle="arc3,rad=-0.2",
                                       facecolor='b',edgecolor='b'))

        start = mplstereonet.stereonet_math.line(20,30)
        end = mplstereonet.stereonet_math.line(10,85)
        dataAx["Hoyo Verde"].annotate("", xy=end, xycoords='data',
                                       xytext=start, textcoords='data',
                                       arrowprops=dict(arrowstyle="simple,head_length=1.0,head_width=1.0,tail_width=0.2",
                                       connectionstyle="arc3,rad=-0.2",
                                       facecolor='b',edgecolor='b'))

        start = mplstereonet.stereonet_math.line(30,50)
        end = mplstereonet.stereonet_math.line(10,90)
        dataAx["Los Cantos"].annotate("", xy=end, xycoords='data',
                                       xytext=start, textcoords='data',
                                       arrowprops=dict(arrowstyle="simple,head_length=1.0,head_width=1.0,tail_width=0.2",
                                       connectionstyle="arc3,rad=-0.05",
                                       facecolor='b',edgecolor='b'))

    #add legend
    ax1 = fig.add_axes([0.28, 0.07, 0.2, 0.1]) #curve A
    ax1.set_xlim(0,1)
    ax1.set_ylim(0,1)
    ax1.text(0.05,0.8,"Legend:",weight="bold")
    c = (0.075,0.6)
    ax1.fill(np.sin(theta)*0.05+c[0],np.cos(theta)*0.05+c[1],
             facecolor='lightgreen', edgecolor='k',linewidth = 0.75, alpha=0.9)
    ax1.text(0.15,0.6,"Dyke strike",verticalalignment='center')
    ax1.scatter(0.075,0.3,color='r',marker='^',s=250,linewidths=1.5,edgecolors='k')
    ax1.text(0.15,0.3,"Radial centre",verticalalignment='center')

    ax1.text(0.6,0.65,"Flow lineation:",verticalalignment='center')
    for y,d,txt in [(0.5,90,"Vertical"),(0.35,45,"45°"),(0.2,0,"Horizontal")]:
        ax1.arrow(0.6,y,0.1,0.0,head_width=0.1,width=0.05,linewidth=1.0,head_length=0.05,length_includes_head=True,
                  edgecolor='k',facecolor=dipcmap(d/90))
        ax1.text(0.75,y,txt,verticalalignment='center')

    ax1.set_xticks([])
    ax1.set_yticks([])


    fig.tight_layout()


    #add square background to on-map stereonets (this is a terrible hack... but works)
    for sub in [gs[0,1],gs[2,3]]:

        #plot white square behind stereonet
        ps = sub.get_position(fig) #ax.get_position(original=False)
        ax2 = fig.add_axes([ps.x0-0.01,ps.y0,ps.width+0.015,ps.height+0.015])
        ax2.set_frame_on(True)
        ax2.set_xticks([])
        ax2.set_yticks([])
        [i.set_linewidth(0.0) for i in ax2.spines.values()]

        #plot lines (on map ax)
        ps = ax2.get_position(fig) #ax.get_position(original=False)
        mapAx.add_artist(mpl.patches.Rectangle((ps.x0,ps.y0),ps.width,ps.height,
                          facecolor='white',edgecolor='k',linewidth=4,
                          zorder=10,transform=fig.transFigure))

    plt.show()
```

```
C:\ProgramData\Anaconda3\lib\site-packages\matplotlib\figure.py:2267: UserWarning: This figure includes Axes that are not compatible
```

```
warnings.warn("This figure includes Axes that are not compatible "
```

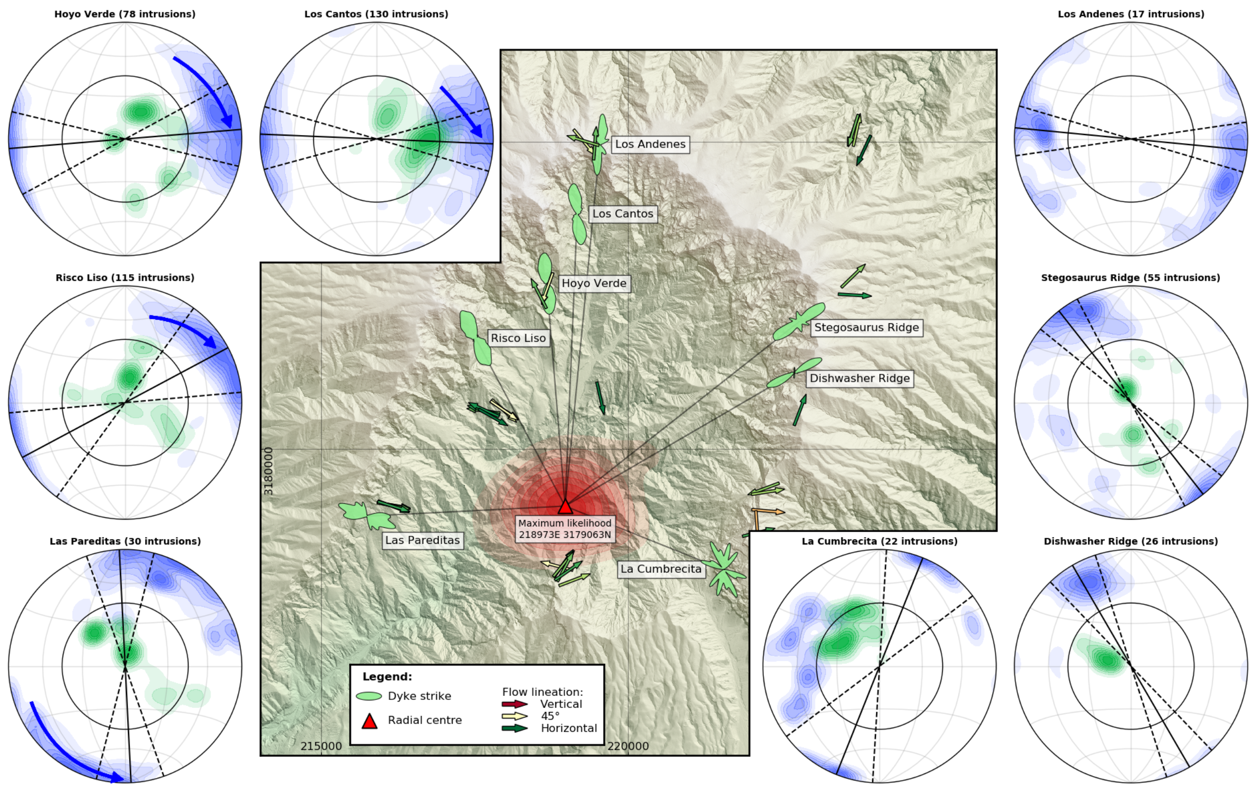


```
In [79]: #save figure
         fig.savefig("Taburiente_map.png",dpi=200)
```

4 Strain

4.1 Estimate circumferential strain

Now that we have estimated the radial centre of the dyke swarm, we can project the dykes onto circumferential scan-lines to estimate the circumfrential strain induced by the dyke swarm.

First, we reload the models so that individul surveys are separate (e.g. there are three surveys at Las Pareditas, two at Hoyo Verde, two at Los Cantos, etc.).

```
In [80]: nm_shallow = [] #non-merged shallow dipping
         nm_steep = [] #non-merged steep dipping
         for i,d in enumerate(data):
             _st,_sh = extractIntrusions( d )
             nm_shallow.append(_sh)
             nm_steep.append(_st)

         clear_output()

         #aggregate intrusions and calculate centers
         nm_agg_steep = []
         nm_agg_shallow = []
         nm_centers = []
         for i in range(len(data)): #loop through field sites
             #create combined SNE objects
             if len(nm_steep[i]) > 0:
                 nm_agg_steep.append( CombinedSNEList("",
```

```
                        [d.SNE for d in nm_steep[i]] ) )
    else:
        nm_agg_steep.append(None)

    if len(nm_shallow[i]) > 0:
        nm_agg_shallow.append( CombinedSNEList("",
                        [d.SNE for d in nm_shallow[i]] ) )
    else:
        nm_agg_shallow.append(None)

    #compute center
    x = np.sum(np.concatenate([sne.pos[0,:] for sne in nm_agg_steep[i].SNEs]))
    y = np.sum(np.concatenate([sne.pos[1,:] for sne in nm_agg_steep[i].SNEs]))
    z = np.sum(np.concatenate([sne.pos[2,:] for sne in nm_agg_steep[i].SNEs]))
    n = np.sum([sne.pos.shape[1] for sne in nm_agg_steep[i].SNEs])
    nm_centers.append( np.array( [x / n, y / n, z / n] ) )
```

Next, scan lines spaced 1-m apart (vertically) are extracted from each of the models. The total extension in the circumfrential direction (sum of dyke apertures projected on to the scan-line) is the calculated, and used to estimate strain for each scan line:

```
In [81]: """
         Project  a 3D vector onto a 2D viewing plane using an orthographic projection
         """
         def proj(P, view, up):
             #ensure vectors are normal
             view = np.array(view)
             view = view / np.linalg.norm(view)
             up = np.array(up)
             up = up / np.linalg.norm(up)

             side = np.cross(view,up)

             #build projection matrix
             M = np.array([side,up,view]).T

             _x = []
             _y = []
             _z = []

             for p in P:
                 U = np.array([p[0],p[1],p[2]])
                 Ut = np.dot(U,M)
                 _x.append(Ut[0])
                 _y.append(Ut[1])
                 _z.append(Ut[2])

             return _x,_y,_z

In [82]: TStrain = [] #tangential strain of each scan line
         RStrain = [] #radial strain of each scan line
         ScanZ = [] #altitude of each scan line
         Gaps = [] #spacing along each scan line
         for n in range(len(data)):
             dykes = nm_steep[n]

             #calculate radial direction
             r = nm_centers[n][0:2] - np.array([mlx,mly])
             pred = np.rad2deg( np.arctan2(r[0],r[1]) )

             #calculate
             minz = min([np.min(sne.pos[2]) for sne in nm_agg_steep[n].SNEs])
             maxz = max([np.max(sne.pos[2]) for sne in nm_agg_steep[n].SNEs])
             pad = 0.1 * (maxz-minz) #calculate pad factor to avoid dodgy results on margins (where digitisation is incomplete)
             minz = int(np.floor(minz+pad))
             maxz = int(np.ceil(maxz-pad))

             #extract slices
             gap = []
             midP = [[],[]]
             colors = []
             t_strain = []
```

```python
r_strain = []
z =[]
for s in range(minz,maxz):
    pos = [] #intersection points
    dilation = []
    D = [] #dykes that are in this slice
    for d in dykes:
        _midP,_trend,_plunge,_thick = d.slice(s-0.5,s+0.5)
        if not _midP is None:
            pos.append([_midP[0],_midP[1],_midP[2]])
            D.append(d)

            #calculate and store dilation vector
            dilation.append( pdf.trendPlunge2Vec(np.deg2rad(_trend),np.rad2deg(_plunge))*_thick )

    #we need  more than ~3 dykes on the scan-line to get a meaningful strain estimat...
    if len(pos) > 3:
        #project and sort
        _x,_y,_z = proj(pos,[r[0],r[1],0],[0,0,1])
        idx = np.argsort(_x)

        #store points for plotting
        midP[0] += _x
        midP[1] += _y
        colors += list(plt.get_cmap("coolwarm")( idx / max(idx) ))

        #calculate and store gaps
        for i in range(1,len(_x)):
            gap.append( _x[idx[i]] - _x[idx[i-1]])

        #calculate change in length in radial,tangential and vertical directions
        _v = np.array([0,0,1]) #vertical unit vector
        _r = np.array([r[0],r[1],0]) #radial unit vector
        _r = _r / np.linalg.norm(_r)
        _t = np.cross(_r,_v) #tangent unit vector
        _t = _t / np.linalg.norm(_t)
        dt = 0 #dilation in the tangent direction
        dr = 0 #dilation in the radial direction
        dv = 0 #dilation in the vertical direction
        for d in dilation:

            dt += np.abs(np.dot(d,_t))
            dr += np.abs(np.dot(d,_r))
            dv += np.abs(np.dot(d,_v))

        #calculate associated strains
        lt = np.max(_x) - np.min(_x) #length of line in tangential direction
        lr = np.max(_z) - np.min(_z) #length of line in the radial direction
        t_strain.append( dt / (lt - dt) ) #strain = change in length / initial length
        r_strain.append( dr / (lr - dr) )
        z.append(s) #store altitude of scan line

#store strain for this model
TStrain.append(t_strain)
RStrain.append(r_strain)
ScanZ.append(z)
Gaps.append(gap)

#skip if not enough measurements
if len(t_strain) < 5:
    continue

#plot slices?
if False:
    plt.figure(figsize=(20,20))

    #plot other dykes in black
    for d in nm_steep[n]:
        for s in [d.upper,d.lower]:
            _x,_y,_z = proj(s.T,[r[0],r[1],0],[0,0,1])
            plt.scatter(_x,_y,color='k',s=0.1,zorder=0)

    #plot shallow dipping in gray
```

```
            for d in nm_shallow[n]:
                for s in [d.upper,d.lower]:
                    _x,_y,_z = proj(s.T,[r[0],r[1],0],[0,0,1])
                    plt.scatter(_x,_y,color='g',s=0.1,zorder=0)

            #plot scanlines
            plt.title(path[n])
            plt.scatter(midP[0],midP[1],color=colors,s=20,lw=1,edgecolors='k')
            plt.gca().set_aspect('equal')
            plt.show()
```

Finally, the strain estimates are merged into broader model groups and confidence intervals (the 10th, 50th and 90th percentiles) calculated for plotting:

```
In [83]: #lists of intrusions
         mTStrain = []
         mRStrain = []
         mScanZ = []
         for ids in groups:
             #loop through datasets and merge strain
             ts = []
             rs = []
             sz = []
             for i in ids:
                 ts += TStrain[i]
                 rs += RStrain[i]
                 sz += ScanZ[i]
             #store merged strain
             mTStrain.append(ts)
             mRStrain.append(rs)
             mScanZ.append(sz)
```

```
In [84]: #extract confidence intervals for strain
         strain = []
         for t in mTStrain:
             if len(t) == 0:
                 strain.append([0,0,0])
             else:
                 strain.append( [np.percentile(t,10)*100,
                                np.percentile(t,50)*100,
                                np.percentile(t,90)*100] )
```

4.2 Estimate vertical strain

We repeat all of the above but using vertical rather than horizontal scan lines, to estimate vertical strain.

```
In [ ]: VStrain = [] #vertical strain of each scan line
        plot = False

        #loop through field sites
        for n in range(len(data)):
            #calculate radial direction
            r = nm_centers[n][0:2] - np.array([mlx,mly])
            pred = np.rad2deg( np.arctan2(r[0],r[1]) )

            #merge shallow and steep datasets and project into plane that is perpendicular to the radial direction
            s_proj = []
            minx = np.inf
            maxx = -np.inf
            _v = r / np.linalg.norm(r) #view direction
            _v = np.array( [_v[0], _v[1], 0] )

            if plot:
                plt.figure(figsize=(10,10))
            for I in [nm_shallow[n],nm_steep[n]]:
                for s in I:
                    _ux,_uy,_uz = proj(s.upper[0:3,:].T,_v,np.array([0,0,1]))
                    _lx,_ly,_lz = proj(s.lower[0:3,:].T,_v,np.array([0,0,1]))
                    _x,_y,_z = proj(s.SNE.pos.T,_v,np.array([0,0,1]))
```

```
            #store min,max _x values
            minx = min( min(np.min(_ux),np.min(_lx)), minx)
            maxx = max( max(np.max(_ux),np.max(_lx)), maxx)

            #store projected data in dyke
            #n.b. to take advantge of the slice function (which slices along common z-values), we put the _x varaible
            #where the _z should be...
            sne = SNEList( _z, _y, _x, s.SNE.trend, s.SNE.plunge, s.SNE.thickness )
            s_proj.append( Dyke("",np.array([_uz,_uy,_ux]),np.array([_uz,_uy,_ux]),sne) )

            if plot:
                plt.scatter(_ux,_uy,color='k',s=0.1)
                plt.scatter(_lx,_ly,color='k',s=0.1)
                plt.scatter(_x,_y,color='r',s=0.1,zorder=2)

    #slice
    midP = [[],[]]
    vS = []
    colors = []
    for x in range( int(minx),int(maxx)):
        pos = [] #intersection points
        dilation = []
        S = [] #sheets that are in this slice
        for s in s_proj:
            _midP,_trend,_plunge,_thick = s.slice(x-0.5,x+0.5)
            if not _midP is None:
                pos.append([_midP[2],_midP[1],_midP[0]]) #store pos back in sensible coords as we've done the slice
                S.append(s)
                dilation.append( pdf.trendPlunge2Vec(np.deg2rad(_trend),np.rad2deg(_plunge))*_thick )

        #calculate strain
        if len(pos) >= 2: #need more than 2 intrusions for valid strain estimate
            _x = np.array(pos).T[0]
            _z = np.array(pos).T[1]

            #sort along z
            idx = np.argsort(_z)
            _x = _x[idx]
            _z = _z[idx]

            #calculate strain
            deltaZ = np.abs(np.sum(np.abs(np.array(dilation).T[2]))) #change in height
            initZ = maxx - minx - deltaZ #initial height
            vS.append(deltaZ / initZ) #vertical strain (% change in height)

            #store points for plotting
            midP[0] += list(_x)
            midP[1] += list(_z)
            colors += [ plt.get_cmap("coolwarm")(vS[-1] / 0.1)] * len(_x)

    #store strain estimates for this model
    VStrain.append(vS)

    #plot?
    if len(vS) > 0 and plot:
        plt.scatter(midP[0],midP[1],color=colors,zorder=4)
        plt.gca().set_aspect('equal')
        plt.title(path[n])
        plt.show()
```

In [86]:
```
#merge into broader groups
mVStrain = []
for ids in groups:
    #loop through datasets and merge strain
    zs = []
    for i in ids:
        zs += VStrain[i]
    #store merged strain
    mVStrain.append(zs)
```

In [87]:
```
#extract confidence intervals for strain
strain2 = []
for t in mVStrain:
```

```
        if len(t) == 0:
            strain2.append([0,0,0])
        else:
            strain2.append( [np.percentile(t,10)*100,
                               np.percentile(t,50)*100,
                               np.percentile(t,90)*100] )
```

4.3 Plot strain rose (Fig. 6)

Plot all the strain estimates on a "rose diagram" to show the distribution of strain through Caldera Taburiente.

```
In [132]: #setup figure canvas
          fig, ax = plt.subplots(1,2,figsize=(13,5)) #fig = plt.figure(figsize=(7,5))

          S = [strain,strain2] #strain to plot
          tSize = [275,550]
          ticks = [[2,4,6,8,10],[1,2,3,4,5]]
          figtitles = ["a. Tangential Strain","b. Vertical Strain"]
          for n,mapAx in enumerate(ax):

              #plot basemap
              mapAx.imshow(base, extent=[topo.xmin,topo.xmax,topo.ymin,topo.ymax],alpha=1.0,zorder=0) #background topo

              #####################################
              #plot likelihood function and maxima
              #####################################
              #maximum likelihood location
              mapAx.scatter(mlx_p,mly_p,color='r',marker='^',s=250,linewidths=1.5,edgecolors='k',zorder=10)

              #which survey areas have strain been calculated for?
              skip = np.sum(np.array(S[n]) != 0,axis=1) == 0
              skip[-1] = True #skip los andenes
              ##################################################
              #plot uav survey location labels
              #################################################
              mapAx.scatter(centers_p[np.logical_not(skip),0],centers_p[np.logical_not(skip),1],color='k',marker='o',s=20)
              for i,pos in enumerate(centers_p):
                  if not skip[i]:
                      if "Las Pareditas" in titles[i]:
                          mapAx.text(pos[0]+300,pos[1]-300,titles[i], verticalalignment='center',horizontalalignment='center',
                                     color='k', bbox=dict(facecolor='white', alpha=0.75))
                      else:
                          mapAx.text(pos[0]+300,pos[1]+300,titles[i], verticalalignment='center',horizontalalignment='center',
                                     color='k', bbox=dict(facecolor='white', alpha=0.75))


              #plot extension rose
              unitSize = tSize[n]
              outer = []
              middle = []
              inner = []
              c = np.array([mlx_p,mly_p]) #centerpoint
              for i,pos in enumerate(centers_p):
                  if not skip[i]:
                      #calculate direction vector (r)
                      r = pos - c
                      r /= np.linalg.norm(r)
                      inner.append(c + r*S[n][i][0]*unitSize)
                      middle.append(c + r*S[n][i][1]*unitSize)
                      outer.append(c + r*S[n][i][2]*unitSize)

                      #plot radial line to this field site
                      mapAx.plot([mlx_p,pos[0]],[mly_p,pos[1]],color='k',alpha=0.75)

              mapAx.plot(np.array(outer).T[0],np.array(outer).T[1],color='b',linestyle=':',alpha=0.75)
              mapAx.plot(np.array(middle).T[0],np.array(middle).T[1],color='blue',linestyle='-',lw=3)
              mapAx.plot(np.array(inner).T[0],np.array(inner).T[1],color='b',linestyle=':',alpha=0.75)
              mapAx.fill( np.concatenate( [np.array(outer).T[0], np.flip( np.array(inner).T[0], axis=0 )] ),
                          np.concatenate( [np.array(outer).T[1], np.flip( np.array(inner).T[1], axis=0 )] ),
                          color='b',alpha=0.15)

              #plot circles
```

```
    _r1 = centers_p[0] - c
    _r2 = centers_p[-2] - c
    _r1 /= np.linalg.norm(_r1)
    _r2 /= np.linalg.norm(_r2)
    _theta1 = np.rad2deg(np.arctan2(_r1[1],_r1[0]))
    _theta2 = np.rad2deg(np.arctan2(_r2[1],_r2[0]))
    for s in ticks[n]:
        mapAx.add_patch( Arc((mlx_p,mly_p),s*unitSize*2,s*unitSize*2,theta1=_theta2,theta2=_theta1,alpha=0.75))
        mapAx.text(mlx_p - _r2[0]*s*unitSize,
                   mly_p - _r2[1]*s*unitSize,
                   "%d " % s, size=11, color='b',weight='bold',
                   verticalalignment='top',horizontalalignment='right' )
    mapAx.text(mlx_p - _r2[0]*5*270,
              mly_p - _r2[1]*5*270-600,
                   "Strain (%)", size=12, color='b', weight='bold',
                   verticalalignment='top',horizontalalignment='center', rotation=_theta1+180 )

    #####################
    #setup map figure
    ####################
    mapAx.set_frame_on(True)
    mapAx.set_xlim(214500,224800)
    mapAx.set_ylim(3177500,3185000)
    mapAx.set_aspect('equal')
    mapAx.set_yticks([3180000])
    mapAx.set_xticks([215000,220000])
    mapAx.set_title(figtitles[n],loc='left')
    for tick in mapAx.get_yticklabels():
        tick.set_rotation('vertical')
        tick.set_verticalalignment('bottom')
    for tick in mapAx.get_xticklabels():
        tick.set_horizontalalignment('left')

    mapAx.tick_params(direction='in',pad=-15)
    mapAx.grid(color='k',alpha=0.4)


#figure legend
fig.tight_layout()
plt.show()
```

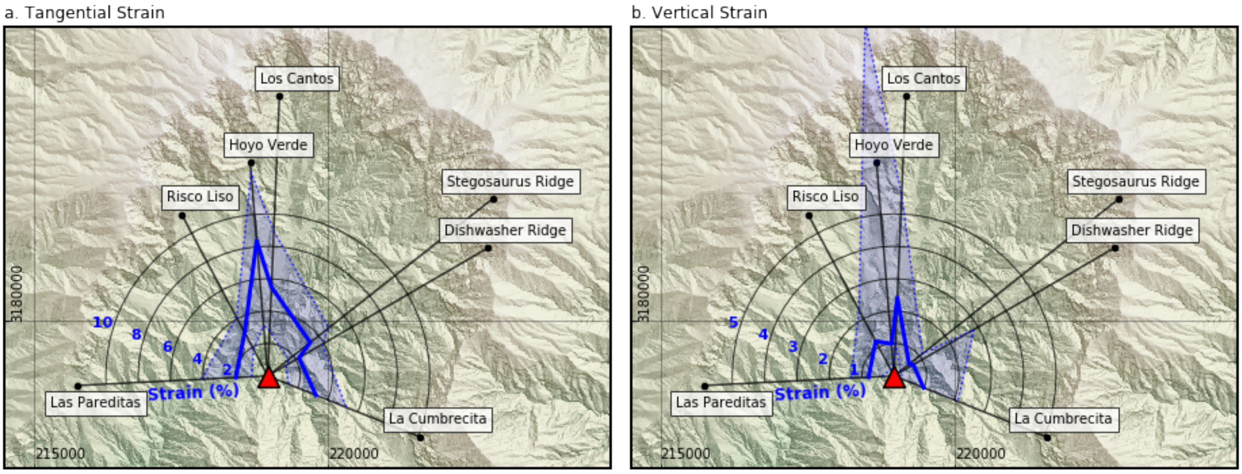


```
In [89]: #save figure
         fig.savefig("strain_rose.png",dpi=300)
```

5 Thickness and aperture

Finally, we analyse the distribution of intrusion thickness (all measurements) and aperture (measurements of the thickest portions of individual intrusions). Although somewhat arbitrary, we define aperture as the 75-90th percentile of all the thickness measurements for each dyke.

In [90]:
```
#each of these lists will contain a KDE object describing the distribution
filtered = []
unfiltered = []
filtered_s = [] #shallow dipping
unfiltered_s = []  #shallow dipping
max_t = 4.5 #maximum allowed thickness... all other measurements considered outliers
for i,t in enumerate(titles):
    m = []
    u = []
    for d in steep[i]:
        #filter points to upper quartile minus outliers (>90th percentile)
        minA = np.percentile(d.SNE.thickness,75)
        maxA = min(np.percentile(d.SNE.thickness,90),max_t)
        #also calculate p dip > 75: CombinedSNEList("unfiltered_shallow",u)
        mask = np.logical_and(d.SNE.thickness > minA, d.SNE.thickness < maxA)

        if np.sum(mask) > 0:
            #add filtered data to SNEList
            m.append( SNEList( d.SNE.pos[0,mask],
                               d.SNE.pos[1,mask],
                               d.SNE.pos[2,mask],
                               d.SNE.trend[mask],
                               d.SNE.plunge[mask],
                               d.SNE.thickness[mask]) )

        #also store unfiltered data (for comparison)
        u.append( d.SNE )

    filtered.append(CombinedSNEList("filtered",m))
    unfiltered.append(CombinedSNEList("unfiltered",u))

    #and the same for shallow dipping sheets
    m = []
    u = []
    for d in shallow[i]:
        #filter points to upper quartile minus outliers (>90th percentile)
        minA = np.percentile(d.SNE.thickness,75)
        maxA = min(np.percentile(d.SNE.thickness,90),max_t)
        mask = np.logical_and(d.SNE.thickness > minA, d.SNE.thickness < maxA)

        #add filtered data to SNEList
        if np.sum(mask) > 0:
            m.append( SNEList( d.SNE.pos[0,mask],
                               d.SNE.pos[1,mask],
                               d.SNE.pos[2,mask],
                               d.SNE.trend[mask],
                               d.SNE.plunge[mask],
                               d.SNE.thickness[mask]) )

        #also store unfiltered data (for comparison)
        u.append( d.SNE )

    if len(m) > 0:
        filtered_s.append(CombinedSNEList("filtered_shallow",m))
    else:
        filtered_s.append(None)

    if len(u) > 0:
        unfiltered_s.append(CombinedSNEList("unfiltered_shallow",u))
    else:
        unfiltered_s.append(None)
#aggregate into SNEList for all dykes
agg = SNEList()
for m in filtered:
    for sne in m.SNEs:
        agg.append( sne )
```

```
agg_uf = SNEList()
for m in unfiltered:
    for sne in m.SNEs:
        agg_uf.append( sne )
```

5.1 Plot thickness distribution

First using unfiltered data:

```
In [91]: plt.figure(figsize=(10,5))
         kdes = []
         gmax = 4.5
         bw = 0.1
         for i,t in enumerate(titles):
             grid,kde = unfiltered[i].getKDE("thick",gmin=0,gmax=gmax,bw=bw)
             plt.plot(grid,kde,label=t+" (%d dykes)" % len(steep[i]),lw=1.2,alpha=0.4)
             kdes.append(kde*len(unfiltered[i].SNEs))

         #also sum kdes and plot
         kdsum = np.sum(kdes,axis=0) #sum
         kdsum /= np.trapz(kdsum,grid) #normalise
         plt.plot(grid,kdsum,label="all",color='k') #plot

         leg = plt.legend(frameon = True)
         frame = leg.get_frame()
         frame.set_facecolor('white')
         frame.set_edgecolor('black')
         ticks = plt.xticks(np.arange(0,10,0.5))
         plt.xlim(0,4.5)
         plt.show()
```

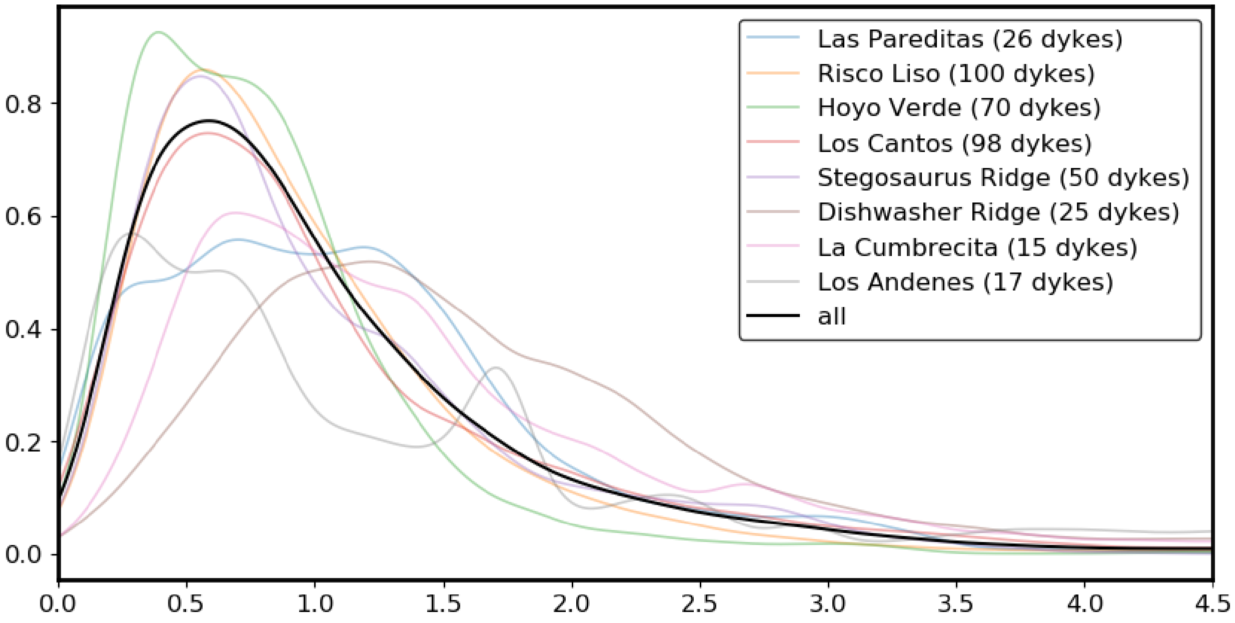

And using the filtered data

```
In [92]: plt.figure(figsize=(10,5))
         kdes = []
         gmax = 15
         bw = 0.1
         for i,t in enumerate(titles):
             grid,kde = filtered[i].getKDE("thick",gmin=0,gmax=gmax,bw=0.1) #n.b. bw=None uses Scott's rule to determine bw
             kde /= np.trapz(kde,grid)
```

```
    plt.plot(grid,kde,label=t+" (%d dykes)" % len(steep[i]),lw=1.2,alpha=0.4)
    kdes.append(kde*len(filtered[i].SNEs))

#also sum kdes and plot
kdsum = np.sum(kdes,axis=0) #sum
kdsum /= np.trapz(kdsum,grid) #normalise
plt.plot(grid,kdsum,label="all",color='k') #plot

leg = plt.legend(frameon = True)
frame = leg.get_frame()
frame.set_facecolor('white')
frame.set_edgecolor('black')
ticks = plt.xticks(np.arange(0,10,0.5))
plt.xlim(0,4.5)

plt.show()
```

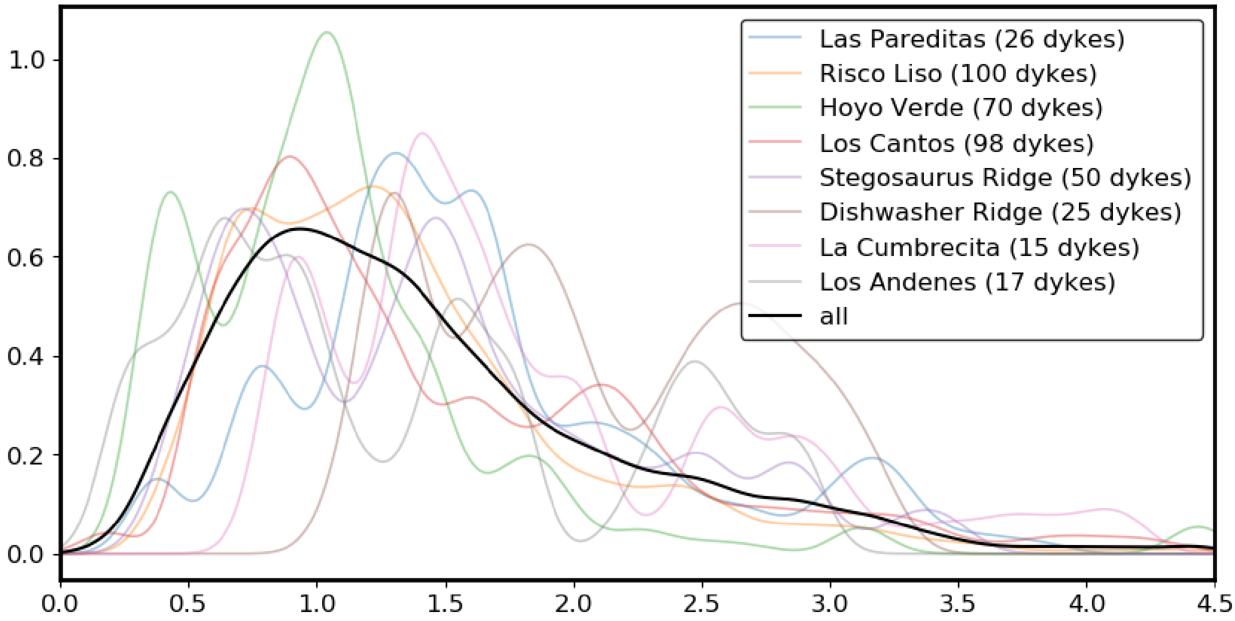

5.2 Bin thickness data by elevation

The apparent relationship between dyke apeture and depth is best shown by binning thickness measurements into 50 m high elevation bins and then using this to construct a violin plot.

```
In [93]: def buildViolins(bins,values,gmax,bw):
             #calculate each violin kde and box-plot
             violins = [] #violin widths
             boxes = [] #box plots quartiles, range and median
             centers = [] #location of each violin/box plot on the x axis
             binsize = bins[1] - bins[0]
             for i,b in enumerate(bins):
                 S = values[i] #SNE List
                 if S is None:
                     continue #skip

                 grid,kde = S.getKDE("thick",gmin=0,gmax=gmax,bw=bw)

                 #calculate cdf
                 cdf = np.zeros(len(kde))
                 for x,t in enumerate(grid):
                     cdf[x] = np.trapz(kde[0:x],grid[0:x])
                 cdf /= np.max(cdf) #max value will be 1
```

```
            #calculate mean from cdf [assumes no negative values!]
            mean = np.trapz(1-cdf,grid)

            #calculate percentiles
            vals = [0] * 5
            for x,t in enumerate(grid):
                for i,threshold in enumerate([0.1,0.25,0.5,0.75,0.9]):
                    if cdf[x] <= threshold:
                        vals[i] = t

            #store
            violins.append({})
            violins[-1]["coords"] = grid
            violins[-1]["vals"] = kde
            violins[-1]['median'] = vals[2]
            violins[-1]['min'] = vals[0]
            violins[-1]['max'] = vals[4]
            violins[-1]['mean'] = mean

            boxes.append({})
            boxes[-1]['q1'] = vals[1]
            boxes[-1]['med'] = vals[2]
            boxes[-1]['q3'] = vals[3]
            boxes[-1]['whislo'] = vals[1] #vals[0]
            boxes[-1]['whishi'] = vals[3] #vals[4]
            boxes[-1]['mean'] = mean

            centers.append(b+binsize/2)

        return centers, violins, boxes
```

```
In [94]: zbase = 1000 #transforms local z to altitude above sea level
         binsize = 50
         gmax=4.5
         vmin = 200 + zbase
         vmax = 1200 + zbase
         bins,values = agg.bin(agg.pos[2]+zbase,binsize,vmin,vmax)
         centers,violins,boxes = buildViolins(bins,values,gmax,bw)
```

```
In [95]: #calculate number of dykes in each model
         ndykes = [0] * len(bins)
         for i,t in enumerate(titles):
             for d in steep[i]:
                 b,v = d.SNE.bin(d.SNE.pos[2]+zbase,binsize,vmin,vmax)
                 for n,_v in enumerate(v):
                     if not _v is None:
                         ndykes[n] += 1
         #drop zeros
         ndykes = [n for n in np.array(ndykes)[np.array(ndykes) > 0]]
```

5.3 Plot thickness and aperture (Fig. 4)

Create a plot of dyke and inclined sheet aperture, thickness and associated change with depth (violin plot) for Fig. 4.

```
In [96]: gmax = 15
         bw = 0.1
         colors = ["dodgerblue","dodgerblue","orangered","orangered"]
         labels = ["i. Dykes (thickness)","ii. Dykes (aperture)","iii. Sheets (thickness)","iv. Sheets (aperture)"]
         style = ['-',':','-',':']
```

```
In [131]: fig,ax = plt.subplots(1,2, figsize=(15,5))
          for n,d in enumerate( [unfiltered,filtered,unfiltered_s,filtered_s] ):
              #evaluate kdes for each model area
              kdes = []
              for i,t in enumerate(titles):
                  if not d[i] is None:
                      grid,kde = d[i].getKDE("thick",gmin=0,gmax=gmax,bw=0.1)
                      kdes.append(kde*len(d[i].SNEs)) #weight each model by how many dykes are in it
```

```
    #combine them
    kdsum = np.sum(kdes,axis=0) #sum
    kdsum /= np.trapz(kdsum,grid) #normalise

    #export KDE for Maxwell model
    if n == 1: #only export dyke aperture
        np.save("aperture/density_combined",np.array([grid,kdsum]))

    #plot
    ax[0].plot(grid,kdsum,label=labels[n],color=colors[n],linestyle=style[n]) #plot

leg = ax[0].legend(frameon = True)
frame = leg.get_frame()
frame.set_facecolor('white')
frame.set_edgecolor('black')

ax[0].set_xlim(0,4.50)
ax[0].set_xlabel("Thickness or Aperture (m)")

ax[0].set_ylabel("Probability density")
ax[0].set_yticks([])

#plot violins
if True:
    vplot = ax[1].violin(violins,centers,widths=binsize*0.6)
    vplot['cmaxes'].set_alpha(0.0)
    vplot['cmins'].set_alpha(0.0)
    vplot['cbars'].set_alpha(0.0)
    for patch in vplot['bodies']:
        patch.set_alpha(0.3)
        patch.set_facecolor('gray')
if True:
    bplot = ax[1].bxp(boxes,centers,widths=binsize*0.75,showmeans=False,showfliers=False)
    for patch in bplot['boxes']:
        #patch.set_facecolor('grey')
        patch.set_alpha(0.75)
        patch.set_linewidth(1.0)
        patch.set_color('r')
    for patch in bplot['whiskers']+bplot['caps']:
        patch.set_alpha(0.75)
        patch.set_linewidth(1.0)
        patch.set_color('r')
    for patch in bplot['medians']:
        patch.set_linewidth(1.25)
        patch.set_color("k")
        #patch.set_edgecolor('gray')
ax[1].set_ylabel("Aperture (m)")
ax[1].set_xlim(vmin,vmax+binsize*0.3)
ax[1].set_ylim(0,4.5)
ax[1].set_xlabel("Elevation (m)")

#plot annotations
ax[1].plot([1200,1350,1850,2200],[1.0,1.0,1.8,1.8],color='k', linewidth=4,linestyle='--',alpha=0.3)
ax[1].plot([1850,2200],[2.5,3.0], color='k', linewidth=4,linestyle='--',alpha=0.3)

#plot number of dykes
ax2 = ax[1].twinx()
ax2.scatter(centers,ndykes,c='b',s=5)
ax2.set_ylabel("Number of Dykes", color='b')
ax2.tick_params(axis='y', labelcolor='b')

ticks = list(np.arange(vmin,vmax+binsize,200))
ax2.set_xticks(ticks)
ax2.set_xticklabels(["%d"%e for e in ticks])
fig.tight_layout()


ax[0].set_title("a. Kernel density estimates of thickness and aperture",x=0.41)
ax[1].set_title("b. Aperture vs elevation",x=0.18)
plt.show()
```

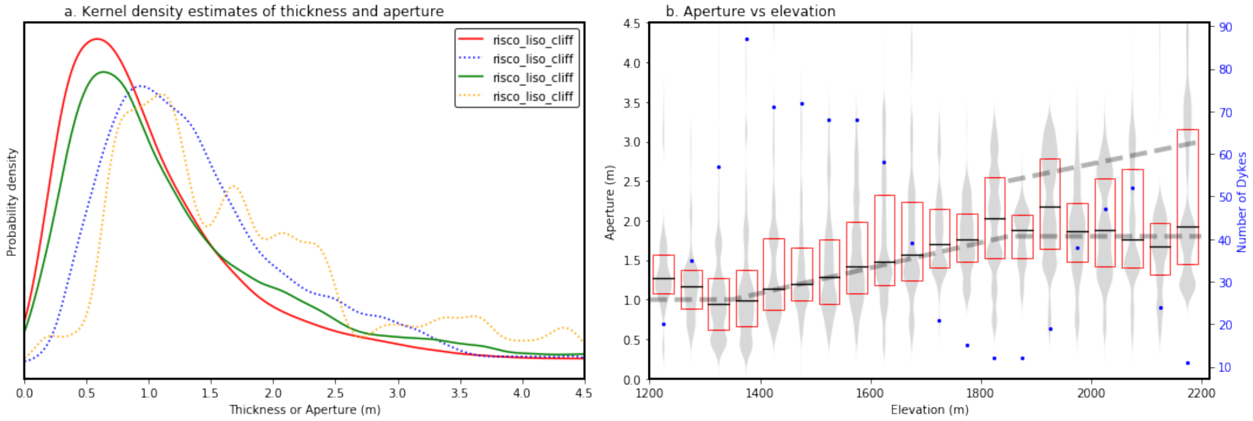


```
In [104]: fig.savefig("thickness.png",dpi=300)
```

5.4 Estimate and plot overpressure (Fig. 5)

Some of the UAV surveys contain dykes that are exposed from tip to tip, meaning the aperture-height scaling relationship can be measured and used to infer magma overpressure. This assumes that: 1. The dykes propagate horizontally 2. They are significantly longer than they are high (i.e. have a blade-shape) 3. Vertical overpressure variations are negligible

Intrusions that are completely exposed are stored in a separate xml node, so we can extract them from the datasets as follows:

```
In [ ]: dip = [] #mean dip of intrusion
        aperture = [] #upper quartile thickness
        span = [] #span vector of the dyke
        pSpan = [] #span vector projected onto vertical plane
        zSpan = [] #vertical distance
        name = [] #name of the dyke
        site = [] #model the dyke is from
        for i,d in enumerate(data):
            #get complete dykes object
            complete = d.filterByName("(Complete Intrusions|complete intrusions)")
            if len(complete) == 0:
                print( "Warning - no complete intrusions found in %s" % path[i])
                continue #no complete intrusions...
            if len(complete) > 1:
                print ("Warning - multiple complete intrusions folders found. These are:")
                print (["%s\n" % c['@name'] for c in complete])
            complete = complete[0] #should only be one...

            #loop through geo-objects in file
            geoObjects = d.filterByKey("GEO_OBJECT",data=complete)


            for g in geoObjects:
                #loop through SNEs belonging to this GeoObject and gather thickness estimates
                SNEs = d.filterByName("SNE_Samples",data=g)
                thickness = []
                nx = []
                ny = []
                nz = []
                trend  = []
                plunge = []
                for s in SNEs:
                    if not "POINTS" in s:
                        continue #empty SNE?

                    #parse structure normals
                    points = s["POINTS"]
```

```python
        _nx = np.fromstring( points["nx"], dtype=np.float, sep=',')
        _ny = np.fromstring( points["ny"], dtype=np.float, sep=',')
        _nz = np.fromstring( points["nz"], dtype=np.float, sep=',')

        #make sure normals all point downwards
        _nx[_nz > 0] *= -1
        _ny[_nz > 0] *= -1
        _nz[_nz > 0] *= -1

        #store
        nx += list(_nx)
        ny += list(_ny)
        nz += list(_nz)

        #parse and store thickness,trend and plunge scalar fields
        thickness += list(np.fromstring( points["thickness"], dtype=np.float, sep=','))
        trend += list(np.fromstring( points["trend"], dtype=np.float, sep=','))
        plunge += list(np.fromstring( points["plunge"], dtype=np.float, sep=','))

    if len(thickness) != 0: #we need at least some thickness estimates...
        #calculate mean orientation by averaging the structure normals
        n_avg = np.array([nx[0],ny[0],nz[0]])
        for _i in range(1,len(nx)):
            n = np.array([nx[_i],ny[_i],nz[_i]])

            #ensure we pick the direction of n that maximises the size of the resultant
            #(as normals can point in either direction)
            if np.dot(n_avg,n) < 0:
                n *= -1

            n_avg += n
        n_avg /= np.linalg.norm(n_avg)
        mean_trend = np.rad2deg(np.arctan2(n_avg[0],n_avg[1]))
        mean_plunge = np.rad2deg(-np.arcsin(n_avg[2]))

        #gather traces and identify span vector (from tip to tip)
        traces = d.filterByKey("TRACE",data=g)
        tips = [] #for now we just assume that the start & end of each trace is a tip...
        for t in traces:
            if not "POINTS" in t:
                continue #empty SNE?

            #parse structure normals
            points = t["POINTS"]
            _x = np.fromstring( points["x"],dtype=np.float,sep=',')
            _y = np.fromstring( points["y"],dtype=np.float,sep=',')
            _z = np.fromstring( points["z"],dtype=np.float,sep=',')

            #store start and end of trace (we don't actually care about the rest)
            tips.append( [_x[0],_y[0],_z[0]] )
            tips.append( [_x[-1],_y[-1],_z[-1]] )

        #find greates span (i.e. maximum distance between tips) by brute force
        tips = np.array(tips)
        maxl = 0
        S = []
        for t1 in tips:
            for t2 in tips:
                l = np.linalg.norm(t1-t2)
                if l > maxl:
                    maxl = l
                    S=t1-t2

        #calculate projection of the span vector onto a vertical plane containing the mean normal vector
        npp = np.cross(n_avg,np.array([0.0,0.0,1.0])) #normal vector to projection plane
        npp = npp / np.linalg.norm(npp) #ensure length=1 - it should be, but floating point errors mean it's not
        ofp = np.dot(S,npp) * npp #calculate component of S perpendicular to the projection plane
        Sp = S - ofp #remove the off-plane component to get S projected onto the projection plane
        assert np.dot(Sp,npp) <= 1e-10, "Error - invalid projection (dot=%f)." % np.dot(Sp,npp) #coplanar?

        #vertical component of span
        zSpan.append( S[2] )
```

```
                #store data
                dip.append(90-mean_plunge)
                aperture.append([np.percentile(thickness,50),np.percentile(thickness,75),np.percentile(thickness,90)])
                span.append(np.linalg.norm(S)) #on-outcrop dyke height
                pSpan.append(np.linalg.norm(Sp)) #projected dyke height
                name.append("%s_%s"%(path[i],g["@name"]))
                site.append(i)

        #convert data arrays to np
        dip = np.array(dip)
        aperture = np.array(aperture)
        span = np.array(span)
        pSpan = np.array(pSpan)
        zSpan = np.array(zSpan)
        site = np.array(site)

        clear_output()
```

```
In [111]: print("A total of %d complete intrusions were found." % len(dip))
```

```
A total of 83 complete intrusions were found.
```

Data from the steeply dipping intrusions (dykes) are then used to estimate the magma overpressures that could explain the observed aspect ratios. A variety of Young's modulii (from 1 - 5 GPa) have been used for these calculations, and the top 25% of pressure estimates treated as outliers (as they result from unusually short/thick dykes).

```
In [118]: #setup figure styles
          plt.style.use(['default'])
          mpl.rcParams['font.size'] = 12
          mpl.rcParams['figure.titleweight'] = 'normal'
          mpl.rcParams['savefig.dpi'] = 350
          mpl.rcParams['axes.spines.bottom'] = True
          mpl.rcParams['axes.spines.left'] = True
          mpl.rcParams['axes.spines.right'] = True
          mpl.rcParams['axes.spines.top'] = True
          plt.rcParams["axes.edgecolor"] = "black"
          plt.rcParams["axes.linewidth"] = 2
          dipcmap = plt.get_cmap("RdYlGn_r")
```

```
In [130]: dipCategories = ["Dykes"]
          drange = [45,90]

          #define elastic properties to use (values used by Becerril et al., 2013, Scientific Reports)
          v = 0.25
          E = np.linspace(1e9,5e9,5) #40 GPa
          pr=2750
          pm=2650
          sd=1e6 #principal stress at surface (1 MPa)
          pe=2.5e6

          #calculate and plot
          gs = mpl.gridspec.GridSpec(1,7)
          fig = plt.figure(figsize=(12,4))
          sax = fig.add_subplot(gs[0,0:3]) #scatter plot axes
          ax = fig.add_subplot(gs[0,3:7]) #KDE axes

          #mask based on dip to get only dykes
          mask=np.logical_and(dip > drange[0], dip < drange[1])
          a = aperture[mask,2] #aperture
          h = pSpan[mask] #we can't use projected span for sills

          #calculate overpressure for scatterplot
          _E = 2.5e9
          P = (a * _E) / (2*h*(1-v**2)) #overpressure in Pa
          P /= 1e6 #convert to MPa

          #plot scatter plot
          thresh = np.percentile(P,75)
```

```
sax.scatter(a[P > thresh],h[P > thresh],facecolors='none',edgecolors=plt.get_cmap("coolwarm")(P[P > thresh]/50))
cbr=sax.scatter(a[P < thresh],h[P < thresh],c=P[P < thresh],cmap="coolwarm",vmin=0,vmax=50)
sax.text(3.3,5.5,"E=2.5 GPa")
sax.text(3.3,4,"v=0.25")
sax.set_yscale('log')
sax.set_xlabel("Aperture (m)")
sax.set_ylabel("Height (m)")
sax.set_title("a. Dyke overpressure",loc="left")
cbar = fig.colorbar(cbr,ax=sax,ticks=[0,50])
cbar.ax.set_ylabel('Overpressure (MPa)',labelpad=-12)

#cbar.ax.set_yticks([0,50])
cbar.ax.tick_params(labelsize=10)

#plot KDEs
for n,_E in enumerate(E):
    #calculate overpressure
    P = (a * _E) / (2*h*(1-v**2)) #overpressure in Pa
    P /= 1e6 #convert to MPa

    #trim top 25%
    P = P[P < np.percentile(P,75)]

    #do KDE
    kde = gaussian_kde(P,bw_method= 7 / np.std(P))
    xs = np.linspace(0,1000,1000)
    pd = kde(xs)

    #plot
    ax.plot(xs,pd,label="E=%d GPa" % (_E / 1e9),color=plt.get_cmap("coolwarm")(_E/max(E)))
    ax.fill(np.append(0,xs),np.append(0,pd),color=plt.get_cmap("coolwarm")(_E/max(E)),alpha=0.15)
    #plot first and last peak
    if (n == 0) or (n == len(E)-1):
        px = xs[np.argmax(pd)]  #get peak
        ax.axvline(px,color=plt.get_cmap("coolwarm")(_E/max(E)))
        ax.annotate("Mode = %d MPa" % px,
                    xy=(px,np.max(pd)),
                    xytext=(px+10,np.max(pd)),
                    arrowprops=dict(facecolor='black', shrink=0.05,width=1,headwidth=7.5,headlength=10),
                    horizontalalignment="left",verticalalignment='center')

#setup KDE plot
ax.set_title("b. Overpressure estimates",loc="left")
ax.set_xlabel("Overpressure (MPa)")
ax.set_ylabel("Frequency")
ax.set_yticks([])
ax.set_xlim(0,100)
ax.set_ylim(0,0.05)
ax.axhline(0,color='k')
ax.legend()

fig.tight_layout()
plt.show()
```

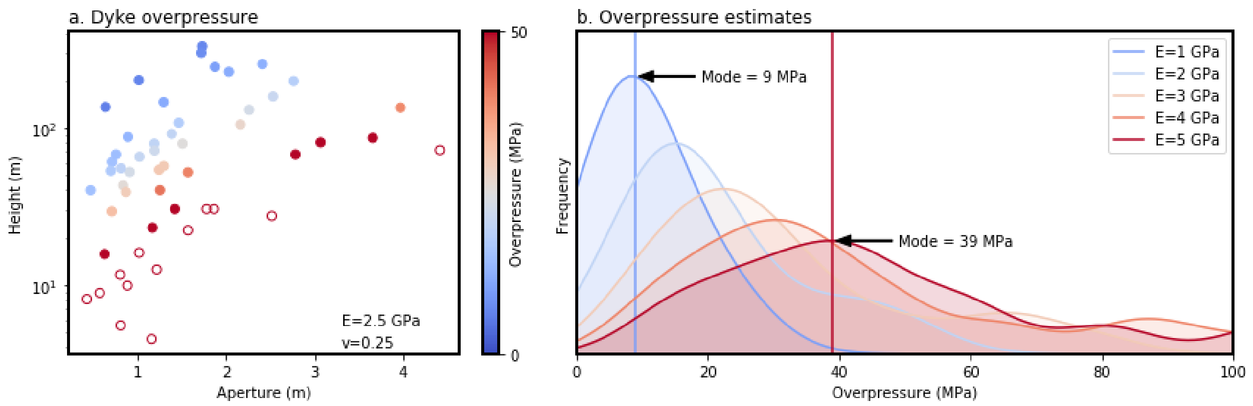

Maxwell model notebook

```
In [137]: import matplotlib.pyplot as plt
          import matplotlib as mpl
          import numpy as np
          import scipy as sp
          from scipy import stats
          from imageio import imread

          %matplotlib inline
          from IPython.display import clear_output

          #hide warnings
          import warnings
          warnings.filterwarnings('ignore')
```

```
In [22]: #setup figure styles
         plt.style.use(['default'])
         mpl.rcParams['font.size'] = 12
         mpl.rcParams['figure.titleweight'] = 'normal'
         mpl.rcParams['savefig.dpi'] = 350
         mpl.rcParams['axes.spines.bottom'] = True
         mpl.rcParams['axes.spines.left'] = True
         mpl.rcParams['axes.spines.right'] = True
         mpl.rcParams['axes.spines.top'] = True
         plt.rcParams["axes.edgecolor"] = "black"
         plt.rcParams["axes.linewidth"] = 2
         dipcmap = plt.get_cmap("RdYlGn_r")
```

```
In [116]: #load field data (KDE of dyke aperture)
          path=r'aperture\\'
          apertureKDE = np.load(path+'density_combined.npy')
```

1 Analytical solution

Implement analytical solution derived previously for the stress evolution over time:

```
In [26]: """
         Model the accomodation of 1d radial dykes by maxwell visco-elasticity

         **Arguments**:
         -E = Young's modulus (Pa)
         -v = poissons ratio
         -mu = viscosity (Pa.sec)
         -Pmax = initial fluid overpressure (Pa)
         -r = the distance from the radial swarm centre the model represents (m)
         -h = the height of the dykes (m)
         -Fd = the rate at which dyking events occur (dykes/year)

         **Keywords**
         -t = the times at which to evaluate the model.
              Default is 0 -> 500kA at 10 year intervals.
         -verbose = True if output should be printed. Default is True.

         **Returns**:
         -stress = the evolution of stress with time
         -strain = the buildup of strain over time
         -aperture = the aperture of dykes at each time
         """
         def MM(E,v,mu,Pmax,r,h,Fd,**kwds):

             #get/calc model args
             secInYear = 60*60*24*365
             L = 2*np.pi*r
```

```
    _t = kwds.get("t",np.linspace(0,0.5e6,0.5e5))
    vb = kwds.get("verbose",True)
    mu = mu / secInYear

    #calculate magic numbers
    k = Fd * 2*h * (1-v**2) / (E * L) #lumped constants
    a = k + 1/mu #this seems to be a magic number
    G = E / (2 * (1+v)) #calculate shear modulus from Young's modulus

    #calculate and print initial aperture
    if vb:
        iA=2*h*(1-v**2)*Pmax/E
        print("Initial dyke aperture = %.2f m" % iA)

    #calculate dyke thickness at equillibrium
    Smax = k*Pmax/a
    eA = 2*h*(1-v**2)*(Pmax-Smax)/E
    if vb:
        print("Equilibrium dyke aperture = %.2f m" % eA)
        print("Equilibrium stress = %.2f MPa" % (Smax / 1e6))
        print("Dyking rate is %d dykes per ka)"%(Fd*1000))

    #calculate stress with time
    _stress = k*(Pmax/a)*(1-np.exp(-2*G*a*_t))

    #calculate strain with time
    num=-k*np.exp(-a*2*G*_t)
    den=2*G*a**2
    _strain = k*Pmax*(num/den-k*_t/a+_t)+(Pmax*k**2)/(2*G*a**2)

    #calculate aperture with time
    _aperture = 2*h*(1-v**2)*(Pmax-_stress)/E

    return _t, _stress, _strain, _aperture
```

1.1 Parmeter optimization

Assuming we can estimate reasonable values for the material properties of our volcano (from other sources), there are three main parameters in the above model that are unknown: h, P_0 and f_d.

These parameters can be directly related to observations of: 1. the maximum dyke aperture prior to a buildup of accomodation stress, 2. the final (equillibrium) dyke aperture and, 3. the final bulk-strain.

Hence, it is possible to solve for the unknown parameters based on field data. Unfortunately the complexity and non-linearity of the equations make it difficult to do this algebraicaly, so we take a numerical approach and use a least-squares solver:

In [27]:
```
"""
Find the maximumum dyke aperture for this set of model params

**Arguments**:
 - see arguments for MM(...).
"""
def Amax(E,v,mu,Pmax,r,h,Fd):
    return 2*h*(1-v**2)*Pmax/E

"""
Find the equillibrium dyke aperture for this set of model params

**Arguments**:
 - see arguments for MM(...).
"""
def Amode(E,v,mu,Pmax,r,h,Fd):
    #get/calc model args
    secInYear = 60*60*24*365
    L = 2*np.pi*r
    mu = mu / secInYear

    #calculate magic numbers
    k = Fd * 2*h * (1-v**2) / (E * L)
    a = k + 1/mu #this seems to be a magic number
```

```
        Smax = k*Pmax/a #calculate the equillibrium stress
        return 2*h*(1-v**2)*(Pmax-Smax)/E #dyke aperture at this stress

    """
    Find the final bulk strain

    **Arguments**:
     - t_final = the time over which the strain developed
                (i.e. how long was the volcano active for?)
     - see arguments for MM(...).
    """
    def Fstrain(t_final,E,v,mu,Pmax,r,h,Fd):
        #get/calc model args
        secInYear = 60*60*24*365
        L = 2*np.pi*r
        mu = mu / secInYear

        #calculate magic numbers
        k = Fd * 2*h * (1-v**2) / (E * L)
        a = k + 1/mu #this seems to be a magic number

        #return final strain
        _t=t_final
        num=-k*np.exp(-a*E*_t)
        den=E*a**2
        return k*Pmax*(num/den-k*_t/a+_t)+(Pmax*k**2)/(E*a**2)
```

```
In [28]: """
    Function to optimize

    **Arguments**:
    -X = a list of model params to be optimised. Should contain
         [Pmax,Fd,h]
    -args = other (fixed) model arguments. Should be
               (E,v,mu,r,t_final,aMax,aMode,strain)
    """
    def func(X,E,v,mu,r,t_final,aMax,aMode,strain):
        Pmax,Fd,h = X

        #check proposed values are valid
        if Fd <= 0:
            return 999999999999999
        if Pmax <= 0:
            return 999999999999999
        if h <= 0:
            return 999999999999999

        #calculate estimates of known values
        _aMax = Amax(E,v,mu,Pmax,r,h,Fd)
        _aMode = Amode(E,v,mu,Pmax,r,h,Fd)
        _strain = Fstrain(t_final,E,v,mu,Pmax,r,h,Fd)

        #return difference
        return [aMax - _aMax,aMode-_aMode,strain-_strain]
```

```
In [128]: #load model diagram for figure
          img = imread('maxwell_model.png')
```

```
In [138]: #define inital value of unknown variables
          Pmax = 60e6 #initial dyke overpressure (Pa)
          Fd = 1/1000
          h = 100 #height of dykes in m
          x0 = (Pmax,Fd,h)

          #define known values
          E = 2.0e9 #young's modulus (Pa)
          v = 0.25 #poisson's ratio
          r = 5000
          t_final = 650e3

          #define field observations to optimize against
          aMax = 3.5
```

```
aMode = 1.0
strain=0.05

#we evaluate a range of values for viscosity as it is very uncertain
mu = [5e22, 4e22, 3e22, 2e22, 1e22] #viscosity (Pa.Year)

fig = plt.figure(figsize=(16,8))
gs = mpl.gridspec.GridSpec(3,10)
ax1 = [fig.add_subplot(gs[0:2,0:5]),fig.add_subplot(gs[0:2,5:10])]
ax2 = [fig.add_subplot(gs[2,0:2]),
       fig.add_subplot(gs[2,2:4]),
       fig.add_subplot(gs[2,4:6]),
       fig.add_subplot(gs[2,6:8]),
       fig.add_subplot(gs[2,8:10])]

#plot model setup
ax1[0].imshow(img)
ax1[0].set_xticks([])
ax1[0].set_yticks([])
ax1[0].axis('off')

cols=['r','orangered','g','b','darkturquoise']
lw = [2,2,2,2,2]
zorder=[1,1,1,1,1]
for i,_mu in enumerate(mu):
    #perform optimization
    args = (E,v,_mu,r,t_final,aMax,aMode,strain)
    x,_ = sp.optimize.leastsq(func,x0,args)

    #print optimum values
    if True:
        print("------Optimized results for Mu = %.1E------" % _mu)
        print("  Initial overpressure is %.1f MPa" % (x[0] / 1e6))
        print("  Dyking rate (Fd) is %E dykes per year (%.1f dykes/ka)"
                 % (x[1],x[1]*1000))
        print("  Dyke height (h) is %d m" % x[2])

    #evaluate
    _t,_stress,_strain,_aperture = MM(E,v,_mu,x[0],r,x[2],x[1],
                                       verbose=False,
                                       t=np.linspace(0,t_final,int(t_final/10)))

    #plot
    title=r"$\mu$=%.0EPa$\cdot$sec, $f_d$=%.1fd/ka, $P_0$=%.1fMPa, h=%dm"%(_mu,
                                                        x[1]*1000,x[0]/1e6,x[2])


    #plot stress
    ax1[1].plot(_t/1000,_stress / 1e6, color=cols[i], lw=lw[i],
                                 zorder=zorder[i], label=title)


    ax1[1].set_ylabel("Accomodation Stress (MPa)")
    ax1[1].set_xlabel("Time (ka)")
    ax1[1].axhline(x[0]/1e6,color=cols[i],linestyle=':')
    ax1[1].text(0,x[0]/1e6+2,r"$P_0$ = %.1f MPa" % (x[0]/1e6),color=cols[i])

    ax2[4-i].hist(_aperture,bins=1000,normed=True,color=cols[i],alpha=0.5)
    ax2[4-i].set_xlabel("Aperture (m)")
    ax2[4-i].text(0.95,0.9,r"$\mu$=%.1E Pa.Sec"%_mu,transform=ax2[4-i].transAxes,horizontalalignment='right')
    ax2[4-i].get_yaxis().set_ticks([])
    ax2[4-i].set_xlim(0,4)

    #add field data
    mask = np.logical_and(apertureKDE[0] > np.min(_aperture),
                          apertureKDE[0] < np.max(_aperture))
    nf = np.trapz(apertureKDE[1][mask],apertureKDE[0][mask])
    ax2[4-i].plot(apertureKDE[0],apertureKDE[1]/nf,color='k',lw=3, label="Field data")
    ax2[4-i].set_ylim(0,np.max(apertureKDE[1]/nf)*2)
    ax2[4-i].axvline(aMode,color='k',linewidth=2,linestyle=':',label="Mode")

#plot timeline
for ax in ax1[1:2]:
```

```
        ax.axvline(650,color='k',linestyle='--')
        ax.axvline(400,color='k',linestyle='--')
        ax.text(0.5,1-0.95,"← Southward migration",transform=ax.transAxes)
        ax.text(0.95,1-0.9,"Cumbre Nueva collapse →",
                horizontalalignment='right', transform=ax.transAxes,)
    ax1[1].legend(loc='upper right')
    ax1[1].set_ylim(0,100)
    ax2[4].legend(loc='center right')

    ax1[0].set_title("a. Maxwell accomodation model",x=0.25)
    ax1[1].set_title("b. Accomodation stress",x=0.15)
    ax2[0].set_title("c. Predicted aperture",x=0.0,horizontalalignment='left')

    #clear text
    clear_output()

    fig.tight_layout()
    fig.show()
```

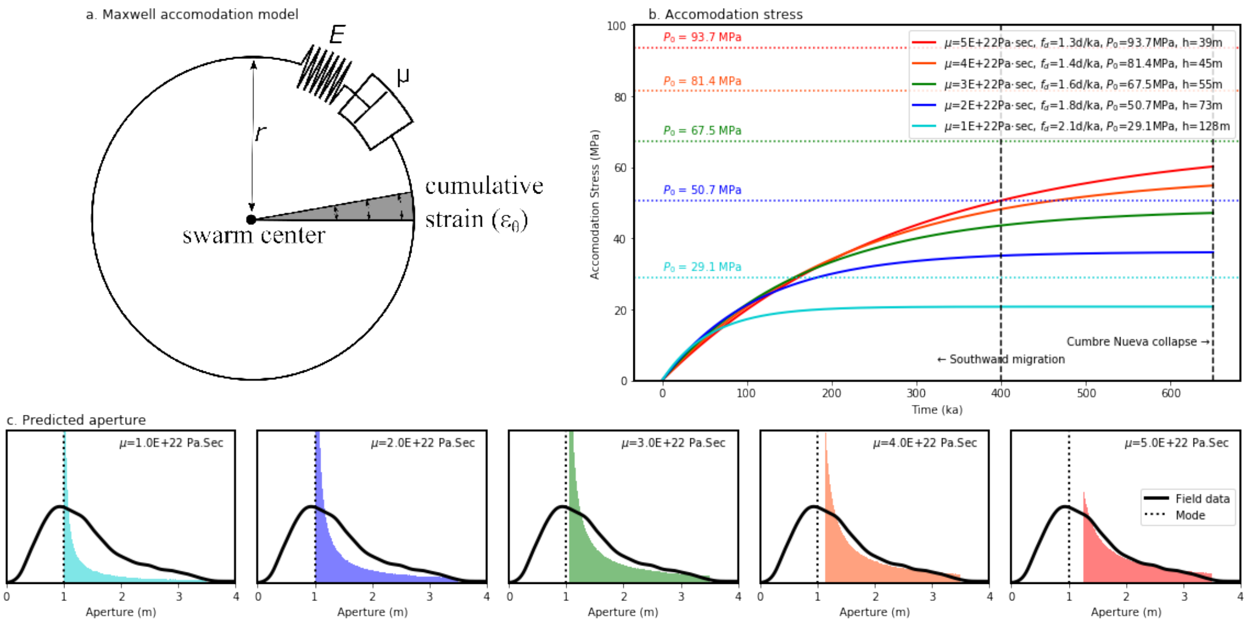

2 Stochastic model

The aperture distributions produced by the previous model have a shape that is generally similar to the observed aperture distribution, however the model cannot produce dykes with apertures less than the asymptote aperture of 1 m (resulting in the "spike" in aperture frequency at 1 m).

To better fit the observed data, we assume that the overpressure in ascending dykes is not uniform (it is very unlikely that it would be!), and add a random element to the model by sampling dyke overpressures from a normal distribution:

$P = N(\mu = P_0, \sigma) - \sigma_{accom}$

Due to the added random element, this can no-longer be solved analytically, so we instead use a numerical solution:

```
In [32]: """
         Model the accomodation of 1d radial dykes by maxwell visco-elasticity
         using a time-stepping numerical approach. This allows the introduction
```

```
of randomly varying magma pressure, determined the the parameter sigma.

**Arguments**:
-E = Young's modulus (Pa)
-v = poissons ratio
-mu = viscosity (Pa.sec)
-Pmax = initial fluid overpressure (Pa)
-r = the distance from the radial swarm centre (m)
-h = the height of the dykes (m)
-Fd = the rate at which dyking events occur (dykes/year)
-sigma = the standard deviation of the normal distribution
         (with mean Pmax) to sample overpressure from.

**Keywords**
-t = the times at which to evaluate the model. Default is 0 -> 500kA
     at 10 year intervals.
-verbose = True if output should be printed. Default is True.

**Returns**:
-stress = the evolution of stress with time
-strain = the buildup of strain over time
-aperture = the aperture of dykes at each time
"""
def MM_numerical(E,v,mu,Pmax,r,h,Fd,sigma,**kwds):
    secInYear = 60*60*24*365 #number of seconds per year
    mu = mu / secInYear #viscosity (Pa.Year)
    L = 2*np.pi*r #length overwhich dykes are emplaced (m)
    vb = kwds.get("verbose",True)
    t = kwds.get("t",np.linspace(0,0.5e6,0.5e5))

    #calculate magic numbers
    k = Fd * 2*h * (1-v**2) / (E * L)
    a = k + 1/mu #this seems to be a magic number
    G = E / (2 * (1+v)) #calculate shear modulus from Young's modulus

    #calculate and print initial aperture
    if vb:
        iA=2*h*(1-v**2)*Pmax/E
        print("Initial dyke aperture = %.2f m" % iA)

    #calculate dyke thickness at equillibrium
    Smax = k*Pmax/a
    eA = 2*h*(1-v**2)*(Pmax-Smax)/E
    if vb:
        print("Equilibrium dyke aperture = %.2f m" % eA)
        print("Equilibrium stress = %.2f MPa" % (Smax / 1e6))
        print("Dyking rate is %d dykes/ka"%(Fd*1000))

    #setup output vars
    stress = np.zeros(len(t))
    strain = np.zeros(len(t))
    aperture = np.zeros(len(t))

    #run model
    ts = t[1] - t[0] #calc timestep
    for i,_t in enumerate(t):
        if i < 1:
            continue #skip first two steps

        #randomly sample a fluid pressure
        P = Pmax + np.random.normal(0,sigma)

        #calculate theoretical aperture of a dyke given the stress
        aperture[i] = 2*h*(1-v**2)*(P-stress[i-1])/E
        if aperture[i] < 0.2: #avoid impossible dykes
            aperture[i] = 0

        #calculate strain this dyke would cause in the host rock
        strain_rate = Fd * aperture[i] / L

        #calculate strain (just as a reference)
        strain[i] = strain[i-1] + strain_rate*ts
```

```
        #calculate next stress
        stress[i] = stress[i-1] + 2*G*(strain_rate - stress[i-1]/mu)*ts

    #return
    return t, stress, strain, aperture
```

2.1 Parameter Optimization

As with the analytical solution, we optimise the model parameters against observations. As the aim of this model is to reproduce the observed dyke aperture distribution, we simply optimise the unknown parameters (the mean and standard deviation of P_0, dyking frequency f_d and height h) by maximizing the overlap between the observed and predicted aperture distributions.

```
In [78]: """
         Function to optimize

         **Arguments**:
         -X = a list of model params to be optimised. Should contain [Pmax,Fd,h,mu,sigma]
         -args = other (fixed) model arguments. Should be (E,v,mu,r,t_final,binnedData,nf)
         """
         def func2(X,E,v,mu,r,t_final,binnedData,nf):
             #Pmax,Fd,h,_mu,sd = X
             Pmax,Fd,h,sd = np.array(X) * np.array(nf)

             #evaluate model numerically
             _t,_stress,_strain,_aperture = MM_numerical(E,v,mu,Pmax,r,h,Fd,sd,
                                                  verbose=False,
                                                  t=np.linspace(0,t_final,1000))

             #calculate normalised histogram and compare with KDE values
             pd, _ = np.histogram(_aperture,bins=50,density=True,range=(0.2,10))

             #return difference between observed distribution and predicted
             #(1 - area of overlap)
             return np.sum( np.abs(pd - binnedData) )

In [139]: #create binned values from measured aperture KDE to compare models to
          bins = np.linspace(0.2,10,50) #bin centers
          bins += (bins[1] - bins[0])/2 #use centers rather than left hand side
          binnedData = sp.interpolate.interp1d(apertureKDE[0],
                                               apertureKDE[1])(bins)

          #set initial values and bounds
          Pmax = 66e6
          Fd = 2.1/1000
          h = 53
          sigma=8.8e6
          mu = 3e22
          E=2.0e9

          #normalisation factors (such that everything is of the order 1-100)
          if False: #re-run the optimisation? (potentially slow)
              nf = (1e6,1e-4,1,1e6)
              x0 = (Pmax,Fd,h,sigma)
              x0 = np.array(x0) / np.array(nf)

              #optimize!
              args = (E,v,mu,r,t_final,binnedData,nf)
              res = sp.optimize.minimize(func2,x0,args,method='Powell', options={"maxiter":1e9})
              Pmax,Fd,h,sigma = np.array(res.x) * np.array(nf)
              if not res.success:
                  print("Error: ", res.message)

              print("\nInitial error was %E" % func2(x0,*args))
              print("Final error is %E" % func2(res.x,*args))

In [121]: #evaluate optimized model
          t,stress,strain,aperture = MM_numerical(E,v,mu,Pmax,r,h,Fd,sigma,
                                          verbose=False,
                                          t=np.linspace(0,t_final,int(t_final/10)))
```

```
In [124]: fig,ax = plt.subplots(1,2, figsize=(14,4))

          #stress
          ax[0].plot(t/1000,stress / 1e6, color='darkred', lw=2)
          ax[0].set_ylabel("Accommodation Stress (MPa)")
          ax[0].set_xlabel("Time (ka)")

          ax[0].yaxis.label.set_color('darkred')
          ax[0].tick_params(axis='y', colors='darkred')

          #hack to get legend/label with params
          title = r"$F_d$=%.1f d/ka, $P_0$=%.1f MPa, h=%d m"%(Fd*1000,Pmax/1e6,h)
          ax[0].scatter([],[],label=title,s=0)
          ax[0].legend(loc='lower right')

          #strain
          ax2 = ax[0].twinx()
          ax2.plot(t/1000,strain*100,color='b',lw=2)
          ax2.set_ylabel("Strain(%)")
          ax2.spines['left'].set_color('darkred')
          ax2.spines['right'].set_color('b')
          ax2.yaxis.label.set_color('b')
          ax2.tick_params(axis='y', colors='b')

          #plot timeline
          ax[0].axvline(650,color='k',linestyle='--')
          ax[0].axvline(400,color='k',linestyle='--')
          ax[0].text(0.62,0.6,"← Southward\n    migration",
                              transform=ax[0].transAxes)
          ax[0].text(0.95,0.45,"Cumbre Nueva    \ncollapse →",
                     horizontalalignment='right', transform=ax[0].transAxes,)

          #aperture
          ax[1].hist(aperture,bins=75,normed=True,range=(0.1,5),
                   color='green',alpha=0.5,
                   label=r"Model prediction")
          ax[1].plot(apertureKDE[0],apertureKDE[1],
                     color='k',lw=3, label="Field data")
          ax[1].set_ylabel("Frequency")
          ax[1].set_xlabel("Aperture (m)")
          ax[1].set_yticks([])
          ax[1].set_xlim(0,5)
          ax[1].legend()

          fig.suptitle(r"Stochastic overpressure ($\sigma$=%.1f MPa)"
                        %(sigma/1e6),
                        y=1.05,x=0.05, horizontalalignment='left')

          fig.tight_layout()
          fig.show()
```

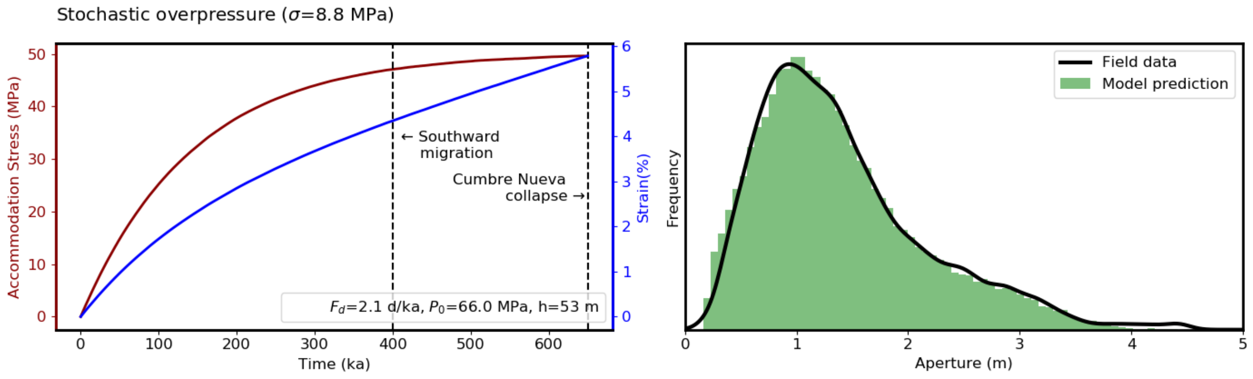

Supplement: Supplementary file 1 — Supplementary Information. [file 41598_2020_74361_MOESM1_ESM.pdf]
